# Species identification and genotyping of *Citrobacter* spp. using genes with high nucleotide diversity

**DOI:** 10.1128/spectrum.03646-25

**Published:** 2026-04-16

**Authors:** Nobuyoshi Yagi, Rin Nashiro, Itaru Hirai

**Affiliations:** 1 Laboratory of Clinical physiology, School of Health Sciences, Faculty of Medicine, University of the Ryukyus 26428, Okinawa, Japan; 2 Laboratory of Microbiology, School of Health Sciences, Faculty of Medicine, University of the Ryukyus 26428, Okinawa, Japan; Zhejiang University School of Medicine Sir Run Run Shaw Hospital, Hangzhou, Zhejiang, China

**Keywords:** nucleotide diversity, genetic classification, *Citrobacter* species

## Abstract

*Citrobacter* species are facultative anaerobic Gram-negative bacilli found in a wide range of habitats. Recently, an increasing number of nosocomial infections caused by *Citrobacter* species have been reported, including strains exhibiting high antimicrobial resistance. In clinical laboratory testing, *Citrobacter* species, such as *Citrobacter freundii* and *Citrobacter braakii*, are often collectively handled as the *Citrobacter freundii* complex. This can hamper species identification and genetic lineage estimation, which are necessary for estimating the distribution mechanism and evaluating the degree of antimicrobial resistance associated with each *Citrobacter* species. In this study, we investigated gene combinations that allow *Citrobacter* species identification and genetic lineage estimation using as few genes as possible. Genome sequences from 18 species were collected from GenBank, yielding a total of 453 genomes. Following gene annotation, nucleotide diversity was calculated for 1,113 genes contained in all the genomes. Genotypes of seven genes with the highest nucleotide diversity were confirmed, and their distributions among *Citrobacter* genomes were investigated. Except for *Citrobacter werkmanii* and *Citrobacter cronae*, the genotype of the highest-nucleotide-diversity gene (groups_3152) showed species-specific distribution, whereas combinations of the top two gene genotypes exhibited genetic lineage-specific distributions. Considering that *Citrobacter werkmanii* and *Citrobacter cronae* genomes are highly similar and difficult to distinguish using conventional genotyping methods, these findings suggest that combinations of the two genes with the highest nucleotide diversity can be used for *Citrobacter* species identification and genetic lineage classification.

**IMPORTANCE:**

The molecular epidemiology of individual *Citrobacter* species remains poorly understood because clinical laboratories often report these organisms collectively as the *Citrobacter freundii* complex. Multilocus sequence typing, which relies on seven conserved housekeeping genes, can also be limited when target loci are absent or deleted in some genomes. In this study, we propose a simple typing strategy based on two genes with high nucleotide diversity (HND) for both species identification and genetic lineage classification of *Citrobacter* species. Using a single HND gene enables species identification, while the combination of two genes allows lineage classification, substantially reducing costs compared with whole-genome sequencing. This approach may facilitate implementation in routine clinical laboratories and promote molecular epidemiological studies of individual *Citrobacter* species.

## INTRODUCTION

Members of the genus *Citrobacter* are facultative anaerobic Gram-negative bacilli detected in human, animal, and environmental samples. Some *Citrobacter* species, including *Citrobacter freundii*, are known to cause nosocomial and opportunistic infections, such as urinary tract infections, meningitis, and sepsis (1–4). Accordingly, *Citrobacter* spp. are regarded as important nosocomial pathogens (5–7). A systematic review by Fonton et al. reported an increasing number of hospitalized patients with *Citrobacter* infections, along with a rising frequency of *Citrobacter* outbreaks in hospitals (8).

Antibiotic treatment of infections caused by *C. freundii*, a major causative species, has sometimes been unsuccessful. This is because *C. freundii* carries an inducible AmpC β-beta lactamase gene, and induction of AmpC β-lactamase nullifies the efficacy of β-lactams, including third-generation cephalosporins (9–11). Currently, higher antimicrobial-resistant (AMR) bacteria, such as extended-spectrum β-lactamase-producing and carbapenem-resistant *Citrobacter* spp., have emerged and spread in many countries (1, 12–14). The emergence of these AMR bacteria has made the treatment of *Citrobacter* infections increasingly difficult.

These trends highlight the need for regular and comprehensive surveillance of *Citrobacter* spp. to monitor infection occurrence and the development of multidrug resistance. From an infection control perspective, it is also important to identify the transmission route and evaluate the clonality of clinical isolates causing nosocomial infections. However, several *Citrobacter* spp., including *Citrobacter braakii*, *C. freundii*, *Citrobacter gillenii*, *Citrobacter murliniae*, *Citrobacter rodentium*, *Citrobacter sedlakii*, *Citrobacter werkmanii*, and *Citrobacter youngae,* are often collectively handled as the *C. freundii* complex. Consequently, the species and clones of causative strains are not always identified, which hinders the assessment of transmission routes and clonality.

Genetic characteristics, such as phylogeny, antimicrobial-resistance genes, virulence genes, and plasmid replicons, are commonly analyzed in bacterial strains obtained from nosocomial infections or outbreaks. These data are essential for surveillance and for estimating the clonality and potential transmission routes. Several analytical methods, such as pulsed-field gene electrophoresis (PFGE) (15), multilocus sequence typing (MLST) (16), matrix-assisted laser desorption/ionization time-of-flight mass spectrometry (17), and whole-genome sequencing (WGS) (18, 19) have been used to characterize pathogenic and AMR clinical isolates.

Certain atypical *Citrobacter* strains have been reported within the clinically important *C. freundii* group (20). These atypical strains can be difficult to identify using standard biochemical tests, which may affect isolate selection for PFGE. Additionally, in the conventional MLST scheme for *Citrobacter* spp., deletions or major mutations in target genes have been reported, which can sometimes prevent the determination of sequence types (STs) (21, 22).

Owing to these limitations, WGS and bioinformatics have become the most widely used methods for genetic characterization. However, WGS remains relatively expensive and laborious and has not yet been adopted as a routine surveillance procedure for pathogenic bacteria in healthcare-associated facilities. Therefore, assessments of clonality and transmission routes are mainly performed in research contexts (23–25).

Furthermore, even if WGS is introduced into clinical testing in the future, the increasing number of samples to be analyzed may impose a substantial burden on computational resources. Therefore, this study aimed to evaluate whether species identification and genetic lineage classification of *Citrobacter* spp. can be achieved using a limited combination of genes. Nucleotide diversity (π) was assessed as a criterion for gene selection. A total of 453 *Citrobacter* genomes were retrieved from the GenBank public database, and core genes shared by all genomes were identified. These genes were ranked based on nucleotide diversity, and the top seven genes with high nucleotide diversity (HND) were selected. As a result, species identification was possible using one of the top two HND genes, while genetic lineage classification of *Citrobacter* spp. was achievable when both genes were used. Furthermore, an independent data set of *Citrobacter* genomes deposited in PubMLST was used to evaluate the relationship between the genetic lineage classification based on the top two HND genes and the results of core genome analysis. The results showed that typing based on these two genes exhibited higher concordance with core genome analysis than the conventional MLST method.

## RESULTS

In this study, 453 *Citrobacter* genomes were collected from GenBank. These genomes belonged to 18 *Citrobacter* spp. (Table S1). Among them, 178 (39.3%) genomes were *C. freundii*, followed by *C. braakii* (81 genomes, 17.9%) and *Citrobacter portucalensis* (55 genomes, 12.1%), as shown in Table 1. STs were determined for the genomes, and species-specific STs were observed in 13 (72.2%) of the 18 *Citrobacter* spp. No identical STs were shared by two or more *Citrobacter* spp. In total, 132 distinct STs were identified in 304 (67.1%) of the 453 genomes. The distribution of STs across *Citrobacter* spp. is shown in Table 1 and Tables S1 and S2.

**TABLE 1:** Genomes examined in this study

			MLST				
			Determined		N.D.		
Species	*N*	%	*N*	%	*N*	%	Major STs
*C. freundii*	178	39.3	157	88.2	21	11.8	22 (31), 116 (12), 150 (8)
*C. braakii*	81	17.9	48	59.3	33	40.7	603 (9), 1107 (7), 1250 (5)
*C. portucalensis*	55	12.1	35	63.6	20	36.4	143 (4), 252 (3), 172 (2)
*C. koseri*	35	7.7	25	71.4	10	28.6	690 (8), 862 (4), 854 (3)
*C. gillenii*	30	6.6	4	13.3	26	86.7	1,115 (2), 699 (1), 1251 (1)
*C. amalonaticus*	18	4.0	12	66.7	6	33.3	710 (3), 712 (3), 716 (3)
*C. cronae*	18	4.0	8	44.4	10	55.6	313 (3), 259 (3), 108 (1)
*C. werkmanii*	8	1.8	4	50.0	4	50.0	104 (3), 108 (1)
*C. youngae*	8	1.8	4	50.0	4	50.0	128 (1), 218 (1), 420 (1), 969 (1)
*C. farmeri*	3	0.7	3	100.0	0	0.0	1,007 (2), 686 (1)
*C. sedlakii*	3	0.7	2	66.7	1	33.3	942 (2)
*C. europaeus*	3	0.7	1	33.3	2	66.7	1,341 (1)
*C. maderi*	3	0.7	1	33.3	2	66.7	1,066 (1)
*C. pasteurii*	3	0.7	0	0.0	3	100.0	–^*a*^
*C. rodentium*	3	0.7	0	0.0	3	100.0	–
*C. eisenhauerii*	2	0.4	0	0.0	2	100.0	–
*C. arseniatis*	1	0.2	0	0.0	1	100.0	–
*C. tructae*	1	0.2	0	0.0	1	100.0	–
Total	453	100.0	304	67.1	149	32.9	

^*a*^ “–” indicates that the sequence type (ST) could not be determined in those species.

The genes present in each *Citrobacter* genome were confirmed (Tables S1 and S3). Across the 453 *Citrobacter* genomes analyzed, a total of 57,628 genes were detected. On average, each genome contained 4,635.2 ± 292.2 genes (mean ± standard deviation [SD]). The median number of detected genes was 4,644, with a maximum and minimum of 7,757 and 3,731 genes, respectively. Of the detected genes, 1,113 (1.9%), 1,357 (2.4%), 316 (0.5%), 2,307 (4.0%), and 52,535 (91.2%) were classified as target core genes, core genes, soft core genes, shell, and cloud genes, respectively (Table S3).

Nucleotide diversity was calculated for the 1,113 target core genes (Table S4). Nucleotide diversity values ranged from 0.0041 to 0.1808. The average nucleotide diversity was 0.0785 ± 0.0284 (mean ± SD), with a median value of 0.0797. The distribution of the calculated nucleotide diversity values is indicated using a box plot. Seven genes were located above the upper whisker of the distribution: groups_3152 (0.1808), *nanK* (0.1782), *iprA* (0.1723), *mipA* (mipA_2, 0.1644), *yehY* (0.1556), *yhcH* (yhcH_2, 0.1552), and *ymdB* (0.1521).

**Fig 1:**
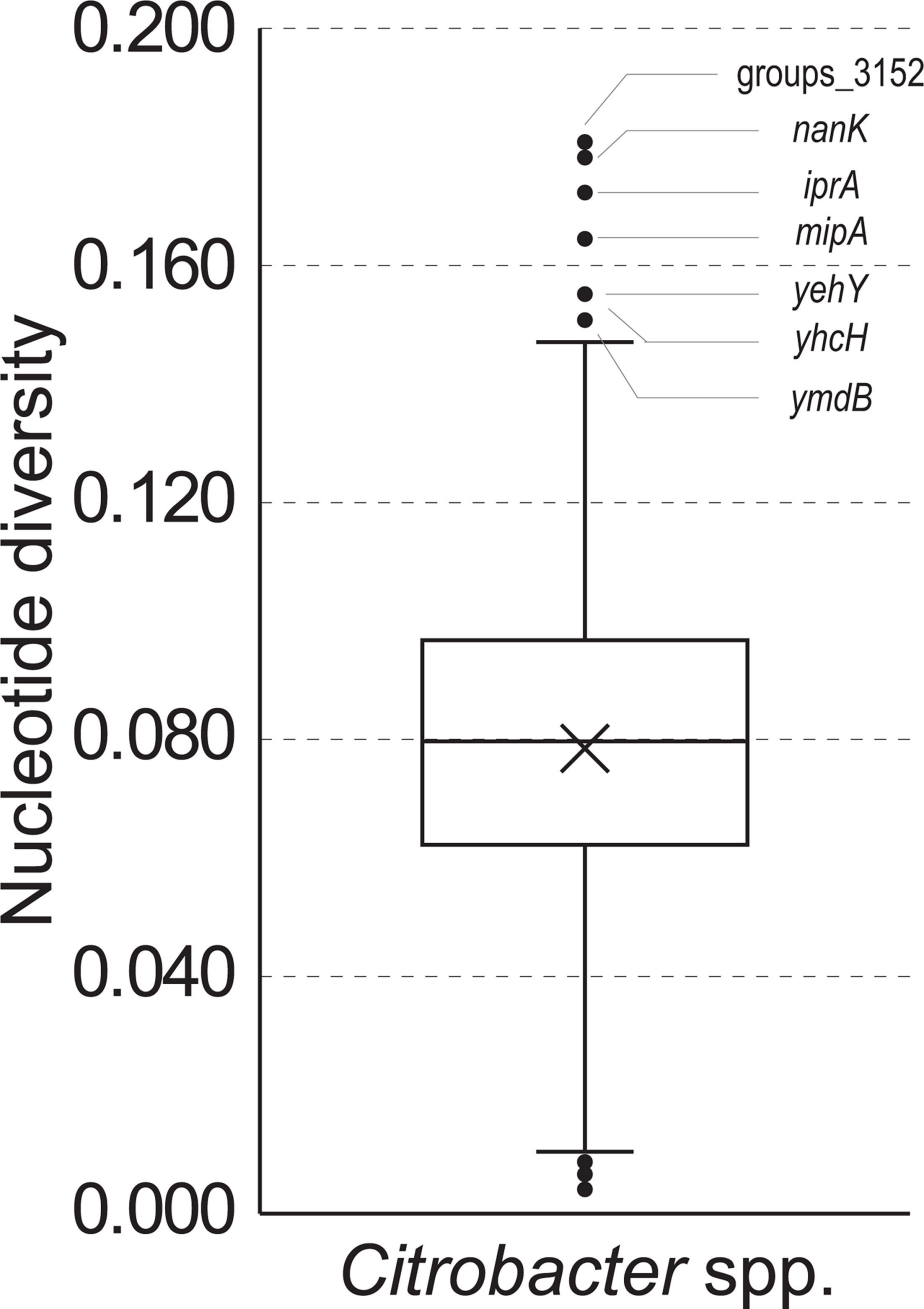
Box plot of nucleotide diversity. Nucleotide diversity of the 1,113 target core genes was calculated. Seven genes showed the highest nucleotide diversity above the upper whisker. These seven HND genes were groups_3152, *nanK*, *iprA*, *mipA* (mipA_2), *yehY*, *yhcH* (yhcH_2), and *ymdB*, with nucleotide diversity rates of 0.1808, 0.1782, 0.1723, 0.1644, 0.1556, 0.1552, and 0.1521, respectively.

We also calculated the detection rate and nucleotide diversity of genes traditionally used for bacterial species identification and multilocus sequence analysis. Detection rates were slightly lower for *rpoB* (99.6%) and *fusA* (99.6%), whereas *recN*, *leuS*, and *pyrG* showed a 100% detection rate. The nucleotide diversity values for these genes were as follows: *recN* (0.1028), *fusA* (0.0278), *leuS* (0.075), *pyrG* (0.044), and *rpoB* (0.0285). Overall, these genes, which are commonly used for bacterial species identification and typing, exhibited lower nucleotide diversity than the top seven HND genes presented in this study. Based on this observation, we evaluated whether the seven HND genes were suitable for species identification and classification of *Citrobacter* spp. (Fig. 1).

Genotypes of the top seven HND genes were identified, yielding 161, 216, 170, 153, 218, 149, and 164 genotypes for groups_3152, *nanK*, *iprA*, *mipA*, *yehY*, *yhcH*, and *ymdB*, respectively. Using these results, the genotypes of all seven HND genes were assigned to each of the 453 *Citrobacter* genomes (Table S1). In total, 237, 255, 262, 271, 272, and 275 genotype combinations were observed for the top two, three, four, five, six, and seven HND genes, respectively.

We next confirmed whether the genotypes of the seven HND genes were distributed in a species-specific manner. Even when using only the top-ranked HND gene (groups_3152), genotypes were largely species-specific. However, three genotypes (groups_3152-13, groups_3152-43, and groups_3152-56) were common between *Citrobacter cronae* and *C. werkmanii* (Table S5). When combinations of the top two HND genes (groups_3152 and *nanK*) were considered, species-specific distribution was observed for all except one genotype combination (groups_3152-43 and *nanK*-70) (Table S6). This genotype combination was detected in two genomes: one *C. cronae* (Genome #317) and one *C. werkmanii* (Genome #413) (Table S1). The STs of these two genomes were not determined in PubMLST, and their genotypes across all seven HND genes were identical. Consequently, these two genomes could not be discriminated using HND genotyping alone.

**Fig 2:**
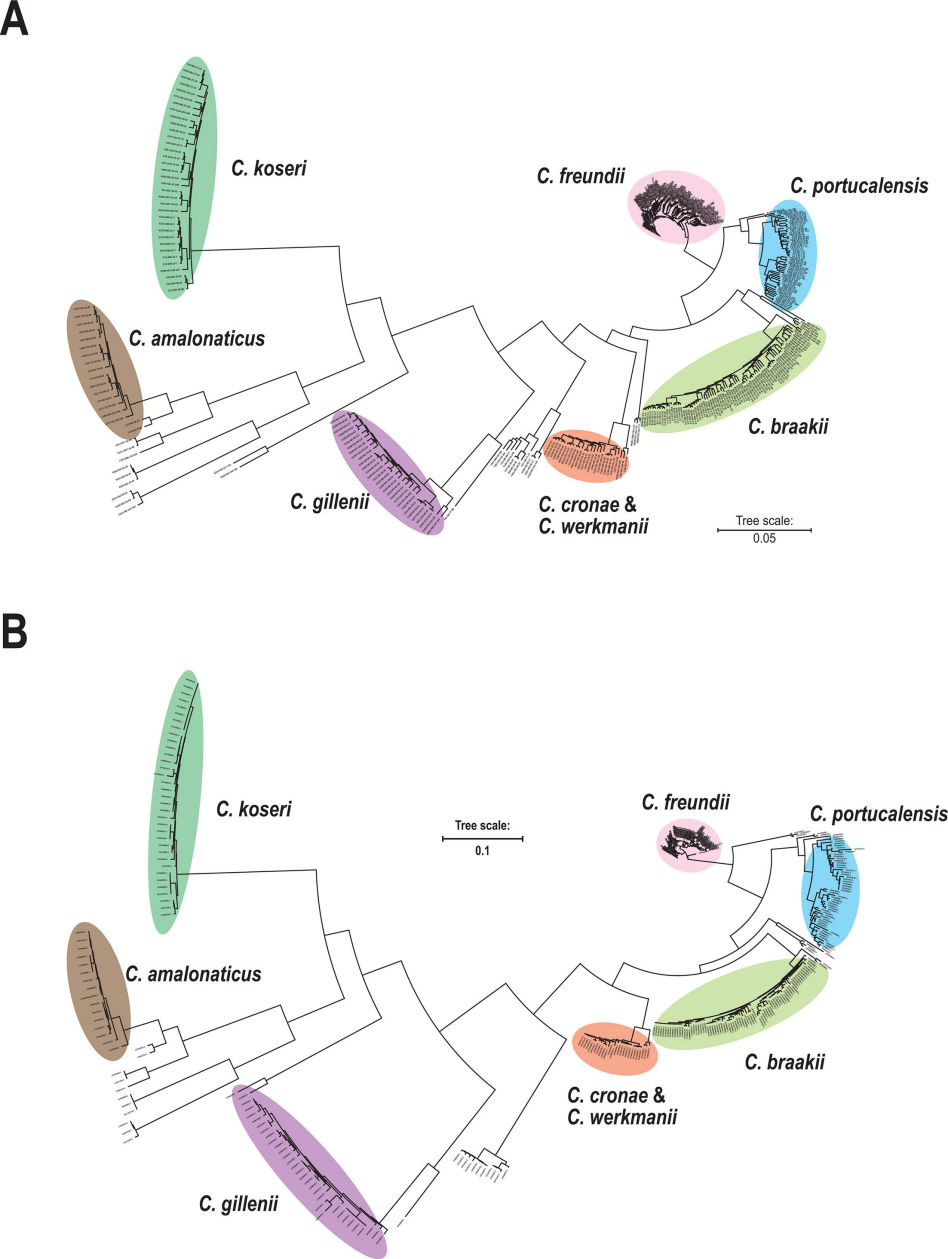
Distribution of *Citrobacter* spp. genomes on phylogenetic trees. (**A**) A phylogenetic tree based on the cgSNP tree. (**B**) A phylogenetic tree based on concatenated sequences of the top two HND genes. Major *Citrobacter* spp. are indicated by color: *Citrobacter freundii* (light red); *Citrobacter braakii* (yellow green); *Citrobacter portucalensis* (blue); *Citrobacter koseri* (green); *Citrobacter gillenii* (pink); *Citrobacter amalonaticus* (brown); *Citrobacter cronae* and *Citrobacter werkmanii* (orange).

To observe phylogenetic relationships, concatenated sequences of the top two HND genes were used to construct phylogenetic trees (HND tree). For comparison, a phylogenetic tree based on core genome single-nucleotide polymorphisms (cgSNPs) (cgSNP tree) was also constructed (Fig. 2). In the cgSNP tree, genomes of the major *Citrobacter* spp., including *C. freundii*, *C. braakii*, *C. portucalensis*, *Citrobacter koseri*, *C. gillenii*, and *Citrobacter amalonaticus,* formed separate branches. In contrast, the genomes of *C. cronae* and *C. werkmanii* were located on the same branch, likely due to insufficient cgSNPs (Fig. 2A). In the HND tree, the *Citrobacter* genomes were similarly distributed into separate branches (Fig. 2B), although the *C. cronae* and *C. werkmanii* genomes were again located on the same branch.

**Fig 3:**
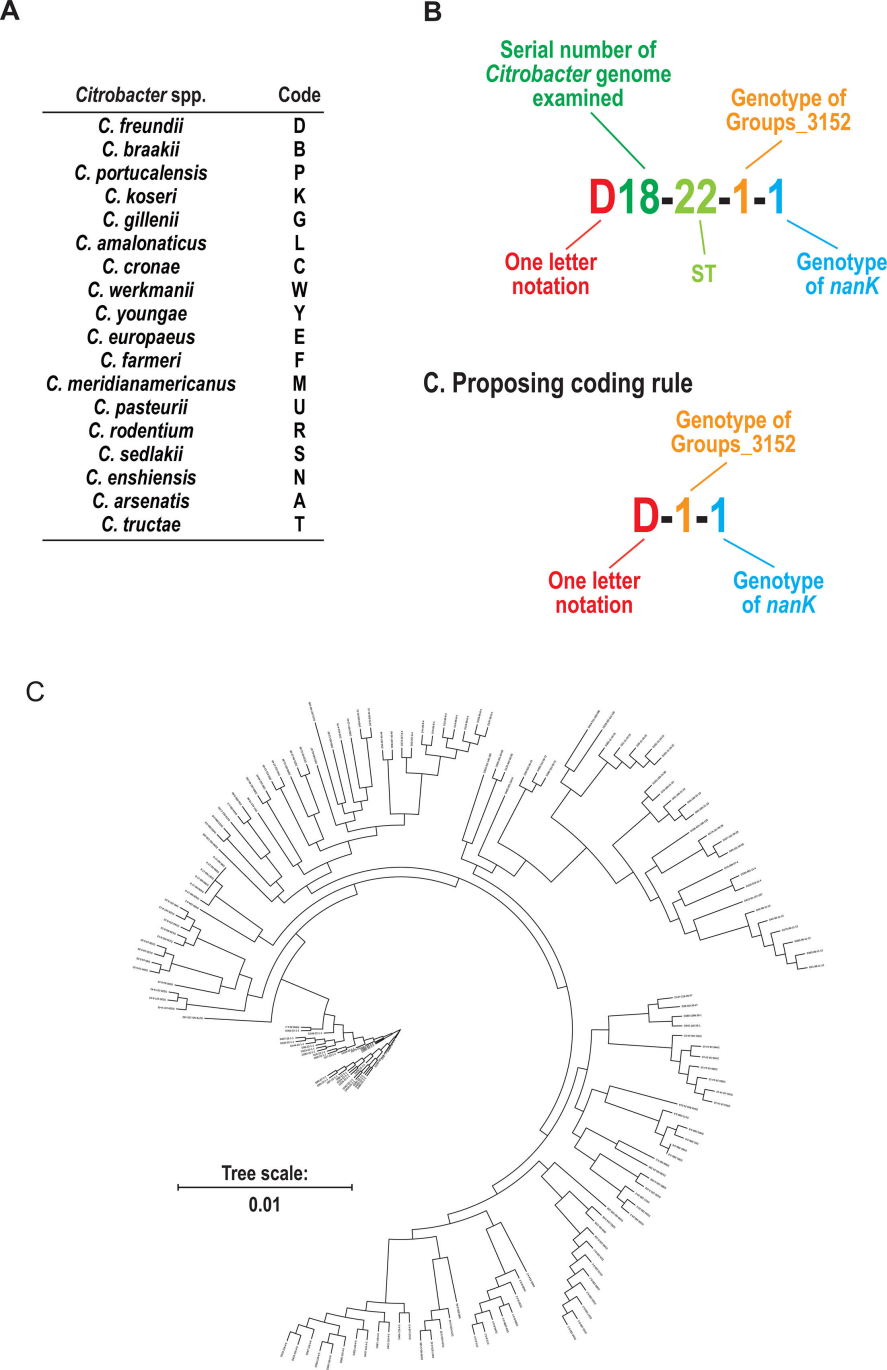
Distribution of *Citrobacter* genome genotypes on the cgSNP tree. To understand the relationship between the genotypes of the top two HND genes (*groups_3152* and *nanK*), sequence type (ST) of each genome, and genome placement in the cgSNP tree, tip labels of the cgSNP phylogeny were converted. Coding system for *Citrobacter* spp. (**A**) and ST, groups_3152, and nanK (**B**) is shown. (**C**) The cgSNP tree with converted tip labels is presented.

To further understand the relationship between the top two HND genes, ST of each genome, and phylogenetic placement, the cgSNP tree branches were examined in detail (Fig. 3; Fig. S1 through S17). Each genome was annotated using a coding system consisting of a one-letter species identifier, genome serial number, the determined ST, and genotypes of the top two HND genes (Fig. S17). In general, genomes sharing the same ST and genotypes as the top two HND genes were closely located within a branch (or rather a twig) of the cgSNP tree. Three major patterns of genome arrangement were observed: (i) genomes with undetermined STs, but located near genomes with defined STs; (ii) genomes located near each other, but with different STs and/or HND gene genotypes; (iii) genomes sharing the same ST (or both undetermined), but with different HND gene genotypes (Table 2).

**TABLE 2:** Summary of observation

Genomes with undetermined ST but located near genome(s) with certain ST on the cgSNP tree						
Coding # of genome^*a*^	Coding # used for comparison^*a*^	Numerical indication of MLST scheme genes^*b*^	Difference of genes in MLST scheme	Numerical indication of the top seven HND genes^*b*^	Difference of the top seven HND genes^*c*^	Presumed ST
D106-ND-1-1	D17-22-1-1	5-15-12-11-20-0-15	lysP deletion	1-1-2-3-1-1-1	None	22
D129-ND-9-13	D140-270-9-13	5-10-12-0-7-11-6	dnaG deletion	9-13-8-49-10-1-23	mipA	270
D133-ND-9-13	D140-270-9-13	5-10-12-0-7-11-6	dnaG deletion	9-13-8-49-10-1-23	mipA	270
D351-ND-14-10	D446-18-14-10	0-10-14-5-17-14-12	arcA deletion	14-10-6-2-189-14-16	yehY	18
K401-ND-15-18	K67-1154-15-18	101-214-0-299-293-178-293	clpX deletion	15-18-26-24-20-30	None	1154
C36-ND-20-81	C208-31-20-22	11-0-18-20-27-15-21	aspC deletion	20-81-15-19-14-17	nanK mutation (G813A)	–^*b*^
C206-ND-20-22	C208-31-20-22	11-24-0-20-27-15-21	clpX deletion	20-22-15-19-14-17	None	31
L251-ND-25-67	L427-563-25-67	115-137-0-184-178-185-194	clpX deletion	25-67-20-15-73-66-12	None	563
D358-ND-12-38	D221-65-12-38	5-16-17-11-20-74-0	mdh deletion	12-38-1-138-191-2-148	iprA mutations (in D221, G403T and deleted after C405)	–
D444-ND-1-2	D34-655-5-80	5-28-167-9-33-5-6	lysP mutations (T406C, A441T, C444T)	1-2-6-5-48-1-9	Groups_3152 mutations (G13A, G135T, G201A, T220C, T276A, G354T, C429G) and nanK mutations (16 nucleotides)	–

Genomes located near genome(s) on the cgSNP tree but their ST and/or genes in the top seven HND genes were different						
Coding # of genome^*a*^	Coding # used for comparison^*a*^	Numerical indication of MLST scheme genes^*b*^	Difference of genes in MLST scheme	Numerical indication of the top seven HND genes^*b*^	Difference of the top seven HND genes^*c*^	
D53-1095-3-2	D390-8-3-2	5-5-336-5-7-5-6	clpX mutation (C342T)	3-2-1-1-11-1-10	None	
D29-257-8-4	D74-95-8-4	5-51-53-6-33-114-15	lysP mutation (T552C)	8-4-1-10-5-4-20	ymdB	
D128-167-9-13	D140-270-9-13	5-10-12-11-7-11-72	mdh mutation (A382G)	9-13-8-1-10-1-23	None	
D153-519-11-4	D156-481-11-4	7-127-10-6-13-11-13	mdh mutation (G303A)	11-4-56-1-65-5-63	None	
D340-112-36-72	D28-622-36-79	5-26-5-54-69-17-15	fadD mutation (G1176A)	36-72-1-6-44-9-38	nanK mutation (T672A)	
D389-1098-28-1	D345-118-28-1	5-64-333-22-33-14-6	clpX mutation (G841T)	28-1-37-1-3-1-29	None	

Genomes with the same ST (or both were ND) and located near genome(s) on the cgSNP tree but their genes in the top seven HND genes were different						
Coding # of genome^*a*^	Coding # used for comparison	Numerical indication of MLST scheme genes^*b*^	Difference of genes in MLST scheme	Numerical indication of the top seven HND genes^*b*^	Difference of the top seven HND genes^*c*^	
D349-22-1-1	D17-22-1-1	5-15-12-11-20-17-15	None	1-1-67-3-16-1-1	iprA mutation (G423A) and yehY; nanK (C763T) and iprA (deletion and mutations after G384)	
D165-150-5-118	D24-150-5-2	10-10-12-9-13-11-10	None	5-2-12-2-22-1-2	mipA	
D259-95-8-4	D197-95-8-4	5-51-53-6-33-5-15	None	8-4-1-114-5-4-8	mipA	
D41-98-11-12	D44-98-11-12	7-54-55-6-60-11-52	None	11-12-78-1-9-9-18	iprA mutation (A251G)	
D42-98-11-12	D44-98-11-12	7-54-55-6-60-11-52	None	11-12-1-73-9-9-18	mipA	
D101-169-21-99	D79-169-21-19	7-80-10-6-56-61-13	None	21-99-27-1-15-1-11	Deletion in nanK (from A250 to A270)	
B173-959-31-34	B148-959-31-115	30-138-140-125-58-147-49	None	31-34-10-32-26-27-46	nanK mutation (T482C)	
B81-ND-38-53	B299-ND-38-53	20-148-0-97-102-208-81	None	38-53-28-43-53-50-40	nanK mutation (G164T)	

^*a*^ The coding scheme follows that described in Figure 3B.

^*b*^ "–" indicates the delimiter between gene types.

^*c*^ Mutated nucleotide positions are indicated only in the top three HND genes.

To validate the utility of HND genotyping, we evaluated groups_3152 and *nanK* using an independent data set of 447 whole-genome sequences obtained from PubMLST. The typing of groups_3152 and *nanK* was performed using BLASTn-based analysis. Among the 447 genomes, *nanK* was not detected in CB00036 and W1_2 (Table S9). In total, 127 and 182 new groups_3152 and *nanK* genotypes, respectively, were identified.

**Fig 4:**
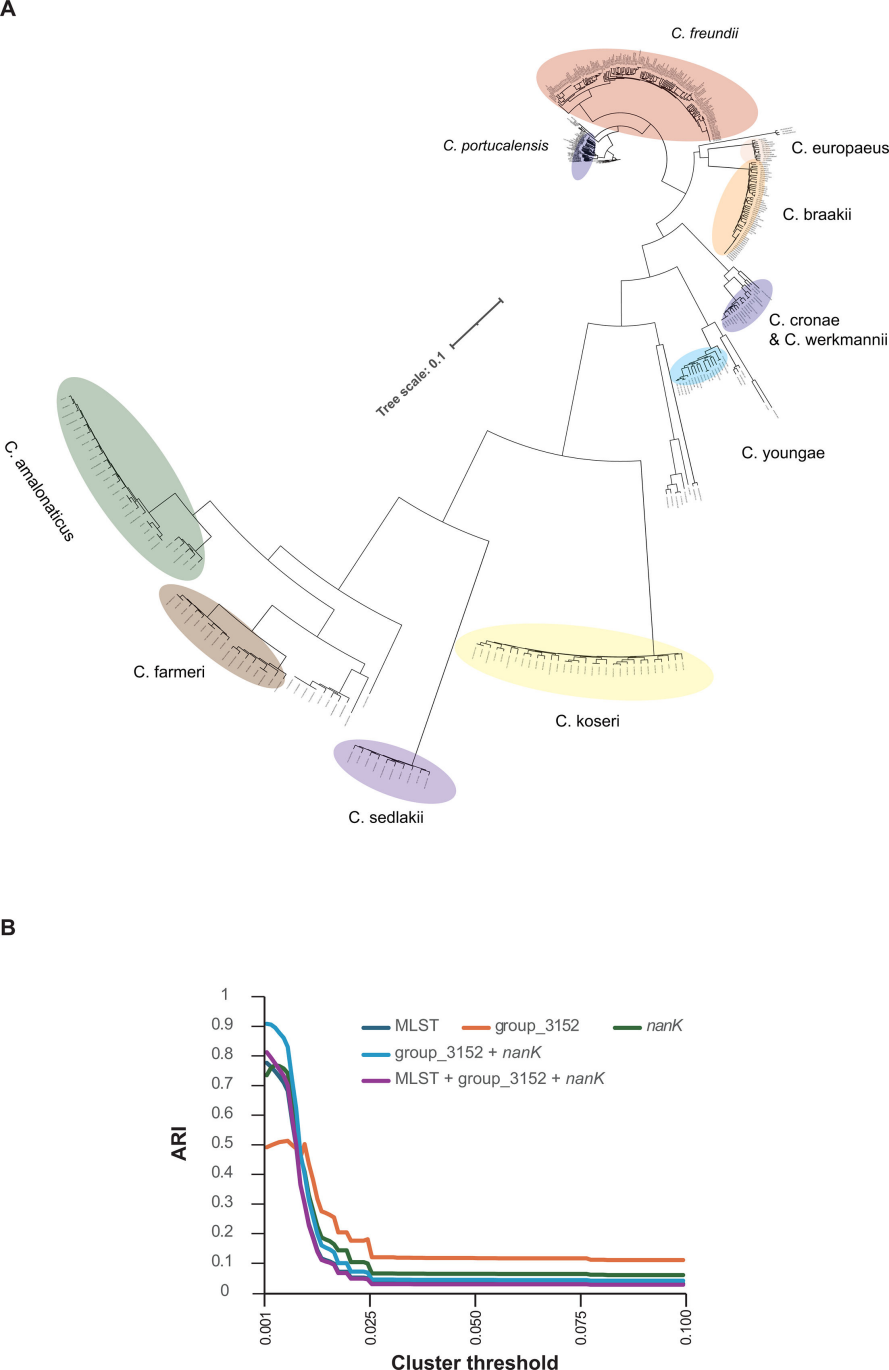
Validation of the utility of two HND genes using 477 *Citrobacter* genomes retrieved from the PubMLST data set. (**A**) A cgSNP tree of 477 *Citrobacter* genomes constructed using Panaroo and IQ-TREE. (**B**) Graph showing ARI values comparing cgSNP-based clustering results with genotyping results based on multilocus sequence typing and the top two HND genes. ARI values were calculated for clustering thresholds ranging from 0.001 to 0.1, as the cluster threshold determines the maximum pairwise distance among all sequences within a cluster and therefore influences the resulting cluster assignments.

A cgSNP tree was constructed using these genome sequences (Fig. 4A), and clustering was performed based on genetic distances within the tree. Genetic distance thresholds ranging from 0.001 to 0.1, in increments of 0.001, were applied (see “Statistical analysis” under Materials and Methods for details). For each threshold, the cgSNP-based cluster IDs were compared with typing results obtained using MLST, as well as groups_3152, *nanK*, and their combinations. The degree of agreement was evaluated using the adjusted rand index (ARI) (Fig. 4B). Across thresholds, the ARI for the groups_3152 + *nanK* combination was consistently higher than that determined by MLST alone. Moreover, the maximum ARI values relative to cgSNP-based clustering were 0.78 for MLST, 0.51 for groups_3152, 0.77 for *nanK*, 0.91 for groups_3152 + *nanK*, and 0.81 for MLST + groups_3152 + *nanK*.

## DISCUSSION

In this study, we selected the top seven HND genes from 1,113 core genes identified in *Citrobacter* spp. genomes retrieved from GenBank. Among these, the two most diverse genes, groups_3152 and *nanK*, were evaluated for their ability to identify *Citrobacter* spp. and discriminate between genetic lineages.

Phylogenetic analysis based on concatenated sequences of these two HND genes revealed a distribution of *Citrobacter* genomes that was highly consistent with that observed in the cgSNP tree (Fig. 2). This result indicates that these two HND genes provide classification power comparable to that of cgSNP analysis, enabling species-level identification for most members of the genus. Specifically, *C. freundii*, *C. braakii*, *C. portucalensis*, *C. koseri*, *C. gillenii*, and *C. amalonaticus* were clearly resolved, whereas *C. werkmanii* and *C. cronae* could not be reliably separated.

Accurate discrimination within the *C. freundii* complex is essential for precise evaluation of antimicrobial resistance dynamics and for understanding species-specific transmission routes in nosocomial infections. Our findings suggest that genotyping based on groups_3152 and *nanK* represents a practical alternative to whole-genome-based approaches for these purposes.

The genomes of *C. werkmanii* and *C. cronae* were intermingled in both HND-based phylogeny and the cgSNP tree (Fig. 2). Consistent with this observation, average nucleotide identity (ANI) analysis revealed values exceeding 96% between these species (Table S7), indicating that genome-based discrimination is intrinsically difficult. A previous study describing *C. cronae* as a novel species similarly reported ANI values of 95.9%–96% relative to that of *C. werkmannii*, concluding that digital DNA–DNA hybridization is required for reliable separation (26).

Taken together, these findings suggest that alternative classification strategies are required for these closely related species and that treating them as a *C. werkmanii* complex may be reasonable in practical applications.

Several genomes clustered on the same branch of the cgSNP tree despite having different or undetermined STs (Fig. 3; Fig. S1–S17). These discrepancies were attributed to missing MLST loci or single-nucleotide mutations affecting allele assignment (Table 2).

Importantly, genomes sharing identical groups_3152 and *nanK* genotypes were consistently clustered together in the cgSNP tree, indicating a shared genetic background. This finding suggests that genotypes of these two HND genes can be used to infer undetermined STs and assess the genetic relatedness among strains, overcoming some inherent limitations of genus-wide MLST schemes.

In *Escherichia coli*, certain STs, such as ST131, function effectively as lineage identifiers. In contrast, MLST applied across the entire genus *Citrobacter* has generated more than 1,000 STs, reducing its utility as an intuitive nomenclature system.

In this context, genotype combinations of groups_3152 and *nanK* provide a simpler and more interpretable alternative. These combinations showed largely species-specific distributions, with the sole exception of the groups_3152-43/nanK-70 genotype shared by *C. werkmanii* and *C. cronae* (Table S6). Independent validation using genomes from the pubMLST database further demonstrated high concordance between HND-based classification and cgSNP clustering, as quantified by the ARI (Fig. 4).

Accordingly, a coding system combining species abbreviations with HND genotypes, optionally supplemented with ST information, may offer a practical framework for molecular epidemiology and transmission analyses.

Although WGS is expected to become increasingly common in clinical microbiology laboratories (27, 28), downstream analyses, such as cgSNP-based phylogenetics, remain labor-intensive. In contrast, HND gene-based genotyping can be rapidly performed using Basic Local Alignment Search Tool (BLAST) against curated databases, enabling efficient species identification and lineage estimation with reduced analytical burden.

Nevertheless, this study has certain limitations. First, publicly available genome data sets are biased toward major *Citrobacter* species, and inclusion of additional genomes from underrepresented species may alter the HND gene ranking. Second, variability in gene annotation, particularly differences in coding sequence boundaries, may influence HND estimates. Standardized reference-based annotations may help mitigate this issue. Because our analysis relied exclusively on public genome data, these limitations could not be directly evaluated using the newly generated isolates.

In conclusion, this study shows that genotyping based on the two highly nucleotide-diverse genes, *groups_3152* and *nanK*, provides species-level identification and lineage discrimination within the genus *Citrobacter* that is largely consistent with cgSNP-based phylogenetic analysis. This approach resolves most *Citrobacter* species examined and represents a practical alternative to whole-genome-based methods for molecular epidemiological analyses. However, considering the aforementioned limitations of our study, future studies must include a broader and more balanced set of isolates, together with standardized annotation strategies, to further evaluate and refine this approach, particularly for closely related species such as those within the *C. werkmanii* complex.

## MATERIALS AND METHODS

### *Citrobacter* genomes and species confirmation

Genomes belonging to the genus *Citrobacter* were retrieved from the GenBank database (29) (last accessed on 24 August 2024) and the PubMLST database (30) (last accessed on 15 December 2025), as listed in Table S1. For GenBank, assemblies labeled as complete genome or chromosome level were selected, and atypical or metagenome-assembly genomes were excluded. For PubMLST, assemblies with a total genome length of ≥4.0 Mb were included in the analysis to exclude incomplete genome sequences. Reference genomes for each *Citrobacter* spp. registered in the RefSeq database (31) were designated as the reference data set for average nucleotide identity analysis using FastANI analysis (32). FastANI was run at the default settings. Each *Citrobacter* genome was compared against the reference data set. Genomes showing >95% ANI with a reference genome were assigned to the corresponding *Citrobacter* spp. MLST was performed for each *Citrobacter* genome using the PubMLST website to determine STs.

### Target core gene extraction

Gene annotation of all collected *Citrobacter* genomes was performed using Prokka (33), and the resulting annotations were used to construct a pan-genome database using PanTA (34). Genes, that is, open reading frames potentially encoding hypothetical proteins identified by Prokka, were grouped by PanTA and labeled sequentially as “groups_1,” “groups_2,” “groups_3,” and so forth. Within the pan-genome database, genes were classified as target core, core, soft core, shell, and cloud genes. These categories were defined as genes detected in 100%, ≥99.0% to <100%, ≥95.0% to <99.0%, ≥15.0% to <95.0%, and ≥0% to <15.0% of the *Citrobacter* genomes, respectively.

### Calculation of nucleotide diversity and genotyping using high nucleotide diversity genes

Nucleotide diversity of the target core genes was calculated using R and RStudio with the “pegas” and “ape” packages. Target core genes were sorted in descending order of nucleotide diversity, and the top seven HND genes were subjected to clustering analysis using VSEARCH software (35). In this study, sequences classified into the same genotype were required to exhibit 100% sequence similarity. Sequences of the top seven HND genes were ranked in descending order according to detection frequency and assigned numbers. Genotypes of the selected genes in each genome were confirmed using BLAST (36).

### Phylogenetic analysis

Sequences of the top seven HND genes in the *Citrobacter* genome were aligned using Multiple Alignment using Fast Fourier Transform (37). Phylogenetic inference was then performed using VeryFastTree (38, 39), and the resulting tree was visualized using Interactive Tree Of Life (iTOL) (40). In addition, concatenated alignments of the selected genes were generated and used for phylogenetic reconstruction, followed by visualization using VeryFastTree and iTOL.

Core genes were identified using Prokka and PanTA as described above. An approximate maximum-likelihood phylogenetic tree was constructed using VeryFastTree with the general time-reversible model with category approximation and 1,000 bootstrap replicates. The resulting tree was visualized using iTOL.

Core genome analysis was performed on genomes retrieved from the PubMLST database. Core genes shared by at least 99% of *Citrobacter* genomes were identified using Panaroo (41) based on Prokka-annotated genomes, and a core-genome alignment was generated. An approximate maximum likelihood phylogenetic tree was then constructed using IQ-TREE (42) with an appropriate nucleotide substitution model and 1,000 bootstrap resamplings.

### Statistical analysis

Phylogenetic clustering was performed using TreeCluster (43) based on the core-genome phylogenetic tree, applying the Max Clade mode with thresholds ranging from 0.001 to 0.1 in increments of 0.001. Singleton clusters, originally labeled as −1, were reassigned specific cluster identifiers (for example, s1, s2, etc.). The ARI was then calculated using R and RStudio with the “mclust” package.

## Data Availability

Gene data sets of groups_3152 and *nanK* generated in this study are publicly available on GitHub (https://github.com/Yagi26/Citrobacter_gene_sets). Genome sequence data analyzed in this study were obtained from public repositories, as listed in Table S1. Other supporting data are available from the corresponding author upon reasonable request.

## SUPPLEMENTAL MATERIAL

Supplemental tables spectrum.03646-25-s0001.xlsx Tables S1 to S9.

Supplemental figures S1 to S5 spectrum.03646-25-s0002.pdf Figures S1 to S5.

Supplemental figures S6 to S10 spectrum.03646-25-s0003.pdf Figures S6 to S10.

Supplemental figures S11 to S14 spectrum.03646-25-s0004.pdf Figures S11 to S14.

Supplemental figures S14 to S17 spectrum.03646-25-s0005.pdf Figures S14 to S17.

ASM does not own the copyrights to Supplemental Material that may be linked to, or accessed through, an article. The authors have granted ASM a non-exclusive, world-wide license to publish the Supplemental Material files. Please contact the corresponding author directly for reuse.
