# Supplementary material for: Species identification and genotyping of Citrobacter spp. using genes with high nucleotide diversity
Source: Microbiol Spectr. 2026 Apr 16;14(6):e03646-25. doi: 10.1128/spectrum.03646-25 (PMC13228044; doi:10.1128/spectrum.03646-25)
Supplement: Supplemental figures S1 to S5 — Figures S1 to S5. [file spectrum.03646-25-s0002.pdf]

Supplementary Figure S1-17

Supplementary Figure S1: Distribution of genomes containing a genotype, groups_3152-1.

Supplementary Figure S2: Distribution of genomes containing a genotype, groups_3152-2.

Supplementary Figure S3: Distribution of genomes containing a genotype, groups_3152-3.

Supplementary Figure S4: Distribution of genomes containing a genotype, groups_3152-4.

Supplementary Figure S5: Distribution of genomes containing a genotype, groups_3152-5.

Supplementary Figure S6: Distribution of genomes containing a genotypes, groups_3152-6, -7, -22 and -37.

Supplementary Figure S7: Distribution of genomes containing a genotype, groups_3152-8.

Supplementary Figure S8: Distribution of genomes containing a genotype, groups_3152-9.

Supplementary Figure S9: Distribution of genomes containing a genotypes, groups_3152-10, -15, -27, -33, -34 and -35.

Supplementary Figure S10: Distribution of genomes containing a genotype, groups_3152-11.

Supplementary Figure S11: Distribution of genomes containing a genotypes, groups_3152-12 and -24.

Supplementary Figure S12: Distribution of genomes containing a genotypes, groups_3152-13, -20, -32 and -43.

Supplementary Figure S13: Distribution of genomes containing a genotypes, groups_3152-14, -17, -19 and -21.

Supplementary Figure S14: Distribution of genomes containing a genotypes, groups_3152-16, -23, -31, -38, -40 and -41.

Supplementary Figure S15: Distribution of genomes containing a genotypes, groups_3152-18 and -29.

Supplementary Figure S16: Distribution of genomes containing a genotypes, groups_3152-25, -26, -30 and -42.

Supplementary Figure S17: Distribution of genomes containing a genotypes, groups_3152-28, -36 and -39.

Supplementary Figure S1

A

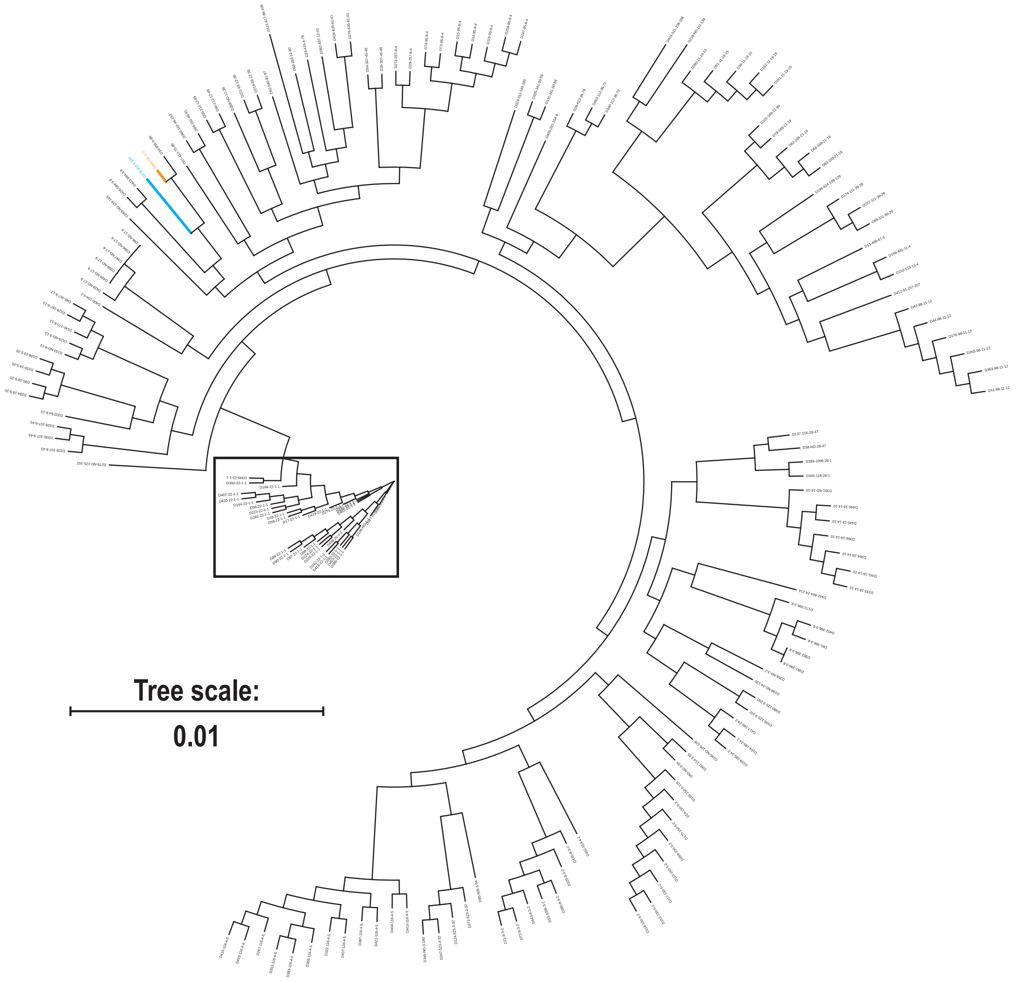

Supplementary Figure S1

B

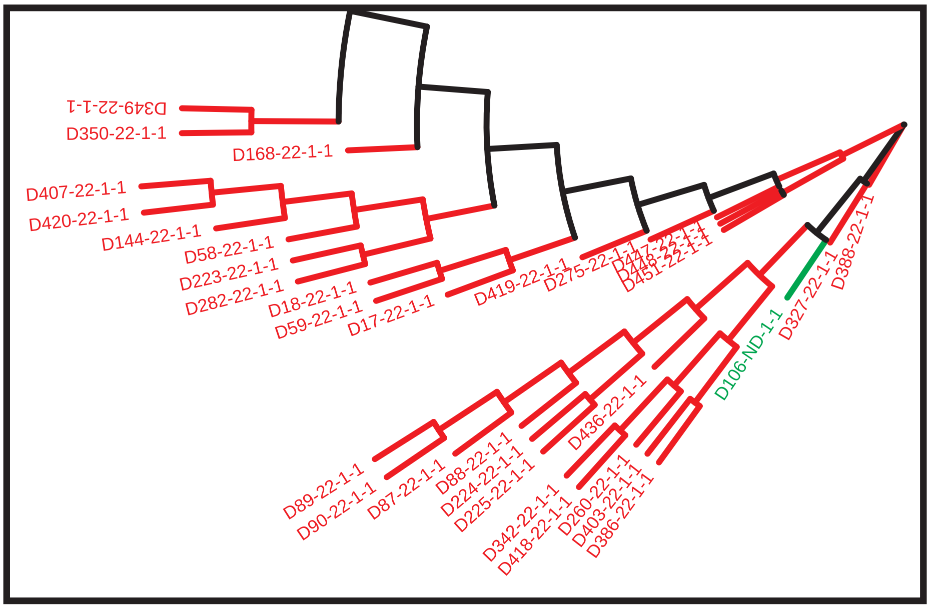

C

			Genotypes of the top seven HND genes.						
Coding	Accession #	ST	groups_3152	*nanK*	*iprA*	*mipA*	*yehY*	*yhcH*	*ymdB*
D17-22-1-1	CP011612.1	22	1	1	2	3	1	1	1
D18-22-1-1	CP011657.1	22	1	1	2	3	1	1	1
D58-22-1-1	CP036435.1	22	1	1	2	3	1	1	1
D59-22-1-1	CP037734.1	22	1	1	2	3	1	1	1
D87-22-1-1	CP047269.1	22	1	1	2	3	1	1	1
D88-22-1-1	CP047273.1	22	1	1	2	3	1	1	1
D89-22-1-1	CP047275.1	22	1	1	2	3	1	1	1
D90-22-1-1	CP047279.1	22	1	1	2	3	1	1	1
D144-22-1-1	CP056365.1	22	1	1	2	3	1	1	1
D223-22-1-1	CP071265.1	22	1	1	2	3	1	1	1
D224-22-1-1	CP071834.1	22	1	1	2	3	1	1	1
D225-22-1-1	CP071907.1	22	1	1	2	3	1	1	1
D260-22-1-1	CP086287.1	22	1	1	2	3	1	1	1
D275-22-1-1	CP098330.1	22	1	1	2	3	1	1	1
D327-22-1-1	CP110775.1	22	1	1	2	3	1	1	1
D342-22-1-1	CP117475.1	22	1	1	2	3	1	1	1
D386-22-1-1	CP137175.1	22	1	1	2	3	1	1	1
D388-22-1-1	CP137183.1	22	1	1	2	3	1	1	1
D403-22-1-1	CP141645.1	22	1	1	2	3	1	1	1
D407-22-1-1	CP145665.1	22	1	1	2	3	1	1	1
D418-22-1-1	CP162975.1	22	1	1	2	3	1	1	1
D419-22-1-1	CP162982.1	22	1	1	2	3	1	1	1
D420-22-1-1	CP163076.1	22	1	1	2	3	1	1	1
D436-22-1-1	LS992175.1	22	1	1	2	3	1	1	1
D447-22-1-1	OW849527.1	22	1	1	2	3	1	1	1
D448-22-1-1	OW967263.1	22	1	1	2	3	1	1	1
D451-22-1-1	OW969875.1	22	1	1	2	3	1	1	1
D168-22-1-1	CP056653.1	22	1	1	2	3	1	98	1
D282-22-1-1	CP099291.1	22	1	1	2	3	164	123	1
D349-22-1-1	CP124809.1	22	1	1	67	3	16	1	1
D350-22-1-1	CP124813.1	22	1	1	67	3	16	1	1
D278-911-1-161	CP099128.1	911	1	161	6	5	161	120	52
D106-ND-1-1	CP054294.1	ND	1	1	2	3	1	1	1
D444-ND-1-2	OW849099.1	ND	1	2	6	5	48	1	9

Supplementary Figure S2

A

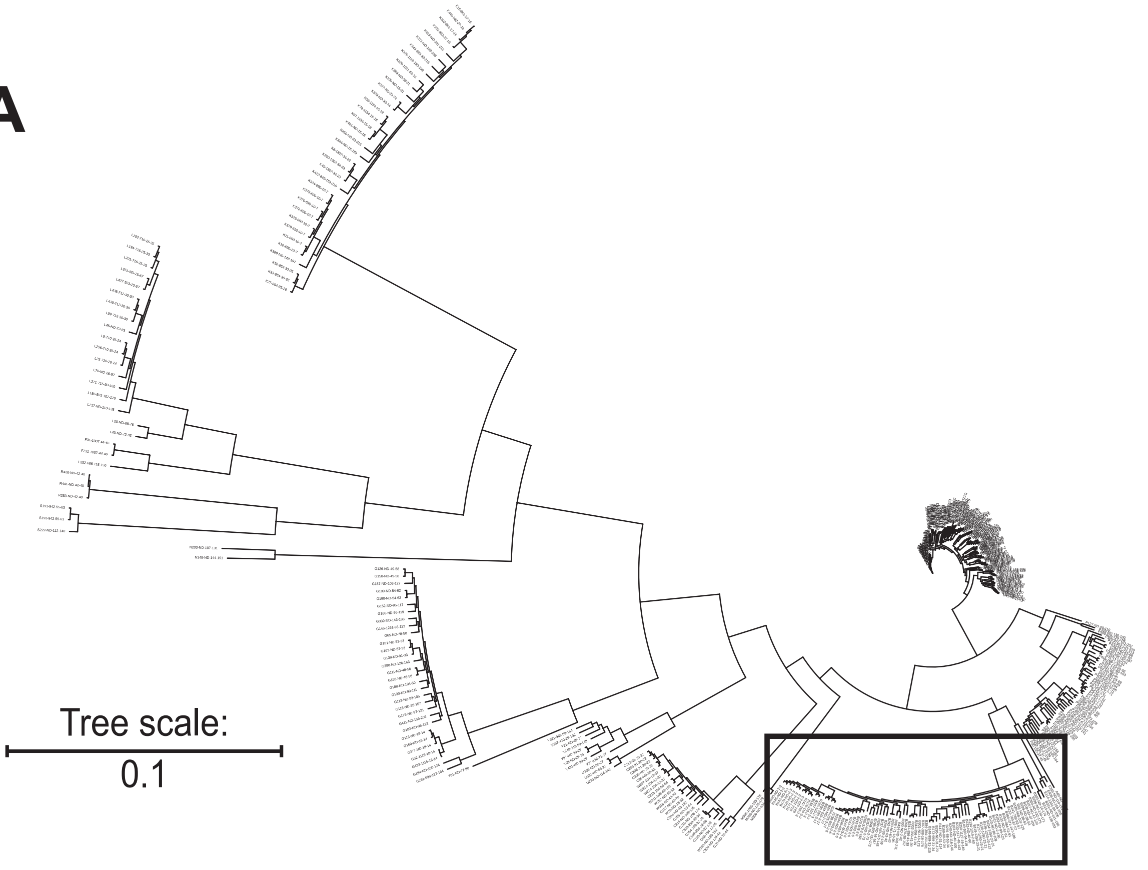

Supplementary Figure S2

B

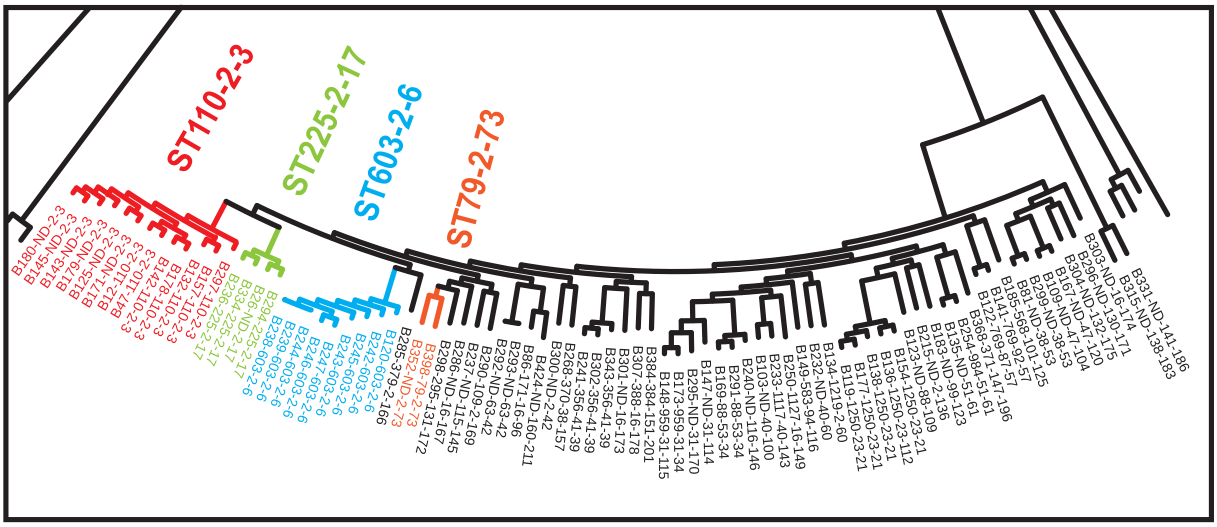

C

			Genotypes of the top seven HND genes.						
Coding #	Accession #	ST	groups_ 3152	*nanK*	*iprA*	*mipA*	*yehY*	*yhcH*	*ymdB*
B12-110-2-3	AP026382.1	110	2	3	4	4	2	3	3
B47-110-2-3	CP026235.1	110	2	3	4	4	2	3	3
B132-110-2-3	CP056251.1	110	2	3	4	4	2	3	3
B142-110-2-3	CP056350.1	110	2	3	4	4	2	3	3
B157-110-2-3	CP056546.1	110	2	3	4	4	2	3	3
B178-110-2-3	CP056888.1	110	2	3	4	4	2	3	3
B297-110-2-3	CP099382.1	110	2	3	4	4	2	3	3
B125-ND-2-3	CP056219.1	ND	2	3	4	4	2	3	3
B143-ND-2-3	CP056361.1	ND	2	3	4	4	2	3	3
B145-ND-2-3	CP056381.1	ND	2	3	4	4	2	3	3
B171-ND-2-3	CP056822.1	ND	2	3	4	4	2	3	3
B179-ND-2-3	CP056896.1	ND	2	3	4	4	2	3	3
B180-ND-2-3	CP056899.1	ND	2	3	4	4	2	3	3
B120-603-2-6	CP056180.1	603	2	6	7	8	6	10	4
B238-603-2-6	CP078595.1	603	2	6	7	8	6	10	4
B239-603-2-6	CP078596.1	603	2	6	7	8	6	10	4
B242-603-2-6	CP078599.1	603	2	6	7	8	6	10	4
B243-603-2-6	CP078600.1	603	2	6	7	8	6	10	4
B244-603-2-6	CP078601.1	603	2	6	7	8	6	10	4
B245-603-2-6	CP078602.1	603	2	6	7	8	6	10	4
B246-603-2-6	CP078603.1	603	2	6	7	8	6	10	4
B247-603-2-6	CP078604.1	603	2	6	7	8	6	10	4
B236-225-2-17	CP078593.1	225	2	17	13	16	23	23	28
B294-225-2-17	CP099378.1	225	2	17	13	16	23	23	28
B334-225-2-17	CP114801.1	225	2	17	13	16	23	23	28
B26-ND-2-17	CP022049.2	ND	2	17	13	16	23	23	28
B300-ND-2-42	CP099386.1	ND	2	42	139	16	170	125	132
B134-1219-2-60	CP056267.1	1219	2	60	100	90	115	95	14
B398-79-2-73	CP138570.1	79	2	73	7	61	202	43	72
B352-ND-2-73	CP126329.1	ND	2	73	17	4	2	43	146
B215-ND-2-136	CP069788.1	ND	2	136	120	11	138	61	3
B285-379-2-166	CP099364.1	379	2	166	63	51	2	61	72
B290-109-2-169	CP099374.1	109	2	169	7	124	168	57	3

Supplementary Figure S3

A

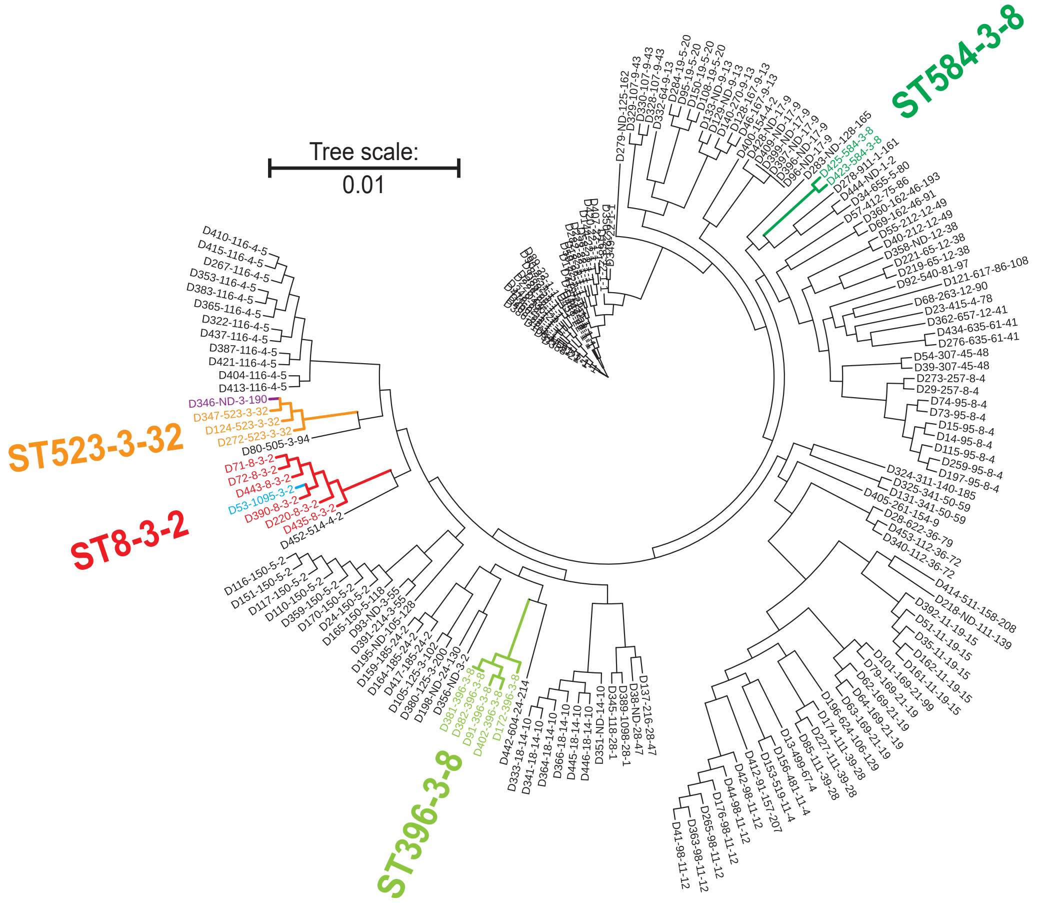

B

Coding #	Accession #	ST	Genotypes of the top seven HND genes.						
			groups_3152	*nanK*	*iprA*	*mipA*	*yehY*	*yhcH*	*ymdB*
D71-8-3-2	CP042478.1	8	3	2	1	1	11	1	10
D72-8-3-2	CP042517.1	8	3	2	1	1	11	1	10
D220-8-3-2	CP070549.1	8	3	2	1	1	11	1	10
D390-8-3-2	CP137201.1	8	3	2	1	1	11	1	10
D435-8-3-2	LR890181.1	8	3	2	1	1	215	1	10
D443-8-3-2	OW849082.1	8	3	2	1	1	11	1	10
D53-1095-3-2	CP032184.1	1095	3	2	1	1	11	1	10
D356-ND-3-2	CP126623.1	ND	3	2	36	2	76	1	147
D91-396-3-8	CP047307.1	396	3	8	18	5	17	2	24
D172-396-3-8	CP056827.1	396	3	8	18	5	17	2	24
D381-396-3-8	CP137123.1	396	3	8	18	5	17	2	24
D382-396-3-8	CP137128.1	396	3	8	18	5	17	2	24
D402-396-3-8	CP140972.1	396	3	8	18	5	17	2	24
D423-584-3-8	CP167050.1	584	3	8	71	2	84	26	48
D425-584-3-8	CP167107.1	584	3	8	71	2	84	26	48
D124-523-3-32	CP056208.1	523	3	32	9	1	3	35	9
D272-523-3-32	CP096921.1	523	3	32	9	1	3	35	9
D347-523-3-32	CP119165.1	523	3	32	9	1	3	35	9
D346-ND-3-190	CP119053.1	ND	3	190	9	1	187	134	9
D391-214-3-55	CP137203.1	214	3	55	1	6	3	51	7
D93-ND-3-55	CP048382.1	ND	3	55	1	6	105	51	2
D80-505-3-94	CP045726.1	505	3	94	91	2	16	14	93
D105-125-3-102	CP054278.1	125	3	102	93	46	57	2	7
D380-125-3-200	CP137117.1	125	3	200	157	46	57	2	7

Supplementary Figure S4

A

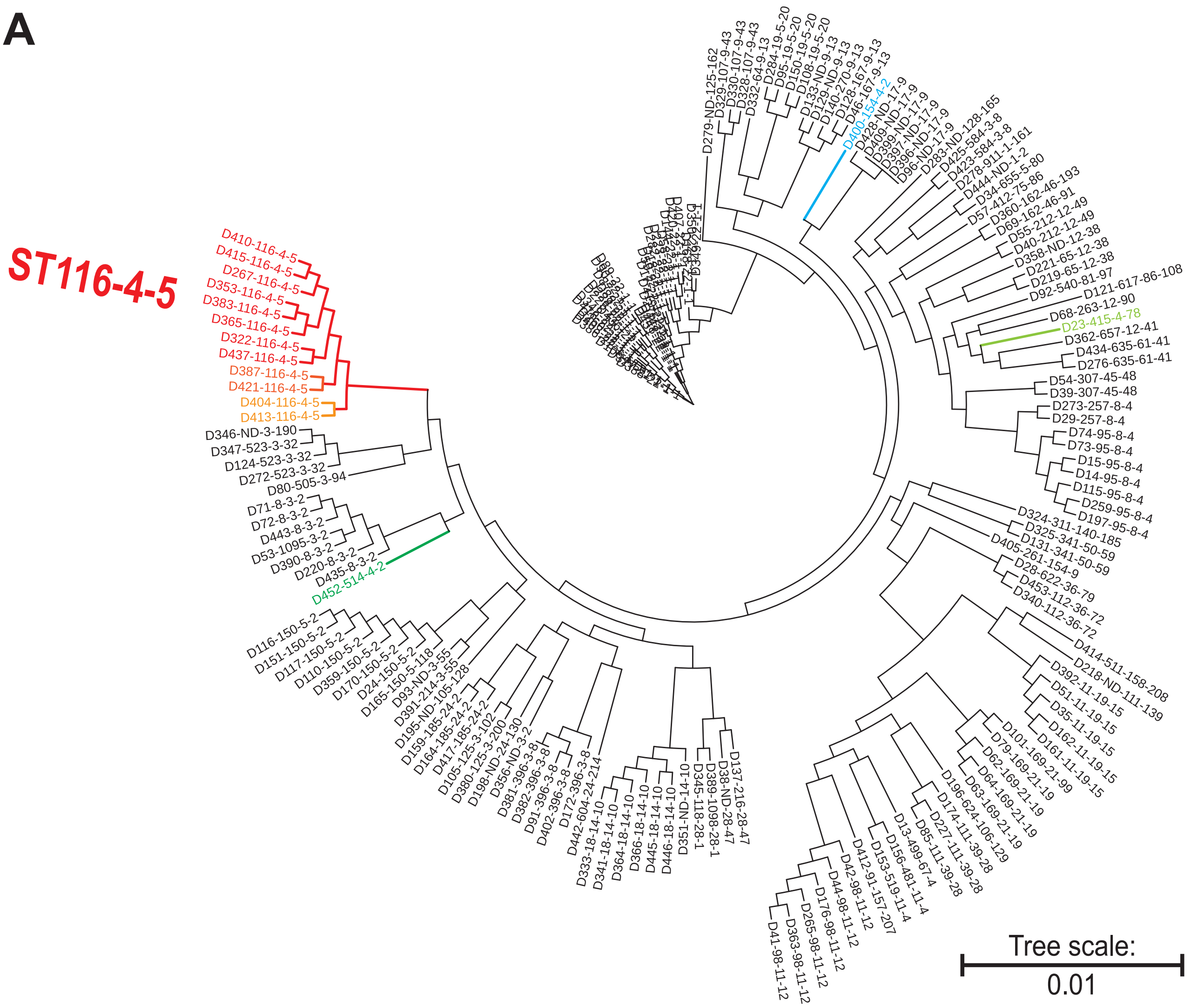

B

			Genotypes of the top seven HND genes.						
Coding #	Accession #	ST	groups_3152	*nanK*	*iprA*	*mipA*	*yehY*	*yhcH*	*ymdB*
D267-116-4-5	CP092493.1	116	4	5	5	2	4	6	2
D322-116-4-5	CP103365.1	116	4	5	5	2	4	6	2
D353-116-4-5	CP126536.1	116	4	5	5	2	4	6	2
D365-116-4-5	CP135622.1	116	4	5	5	2	4	6	2
D383-116-4-5	CP137133.1	116	4	5	5	2	4	6	2
D410-116-4-5	CP150622.1	116	4	5	5	2	4	6	2
D415-116-4-5	CP155122.1	116	4	5	5	2	4	6	2
D437-116-4-5	LS992183.1	116	4	5	5	2	4	6	2
D387-116-4-5	CP137179.1	116	4	5	5	2	4	6	78
D421-116-4-5	CP165768.1	116	4	5	5	2	4	6	78
D404-116-4-5	CP142904.1	116	4	5	5	2	16	6	2
D413-116-4-5	CP151860.1	116	4	5	5	2	16	6	2
D400-154-4-2	CP139850.1	154	4	2	44	146	10	142	9
D23-415-4-78	CP016762.1	415	4	78	73	70	88	2	82
D452-514-4-2	OW969904.1	514	4	2	1	6	49	1	164

Supplementary Figure S5

A

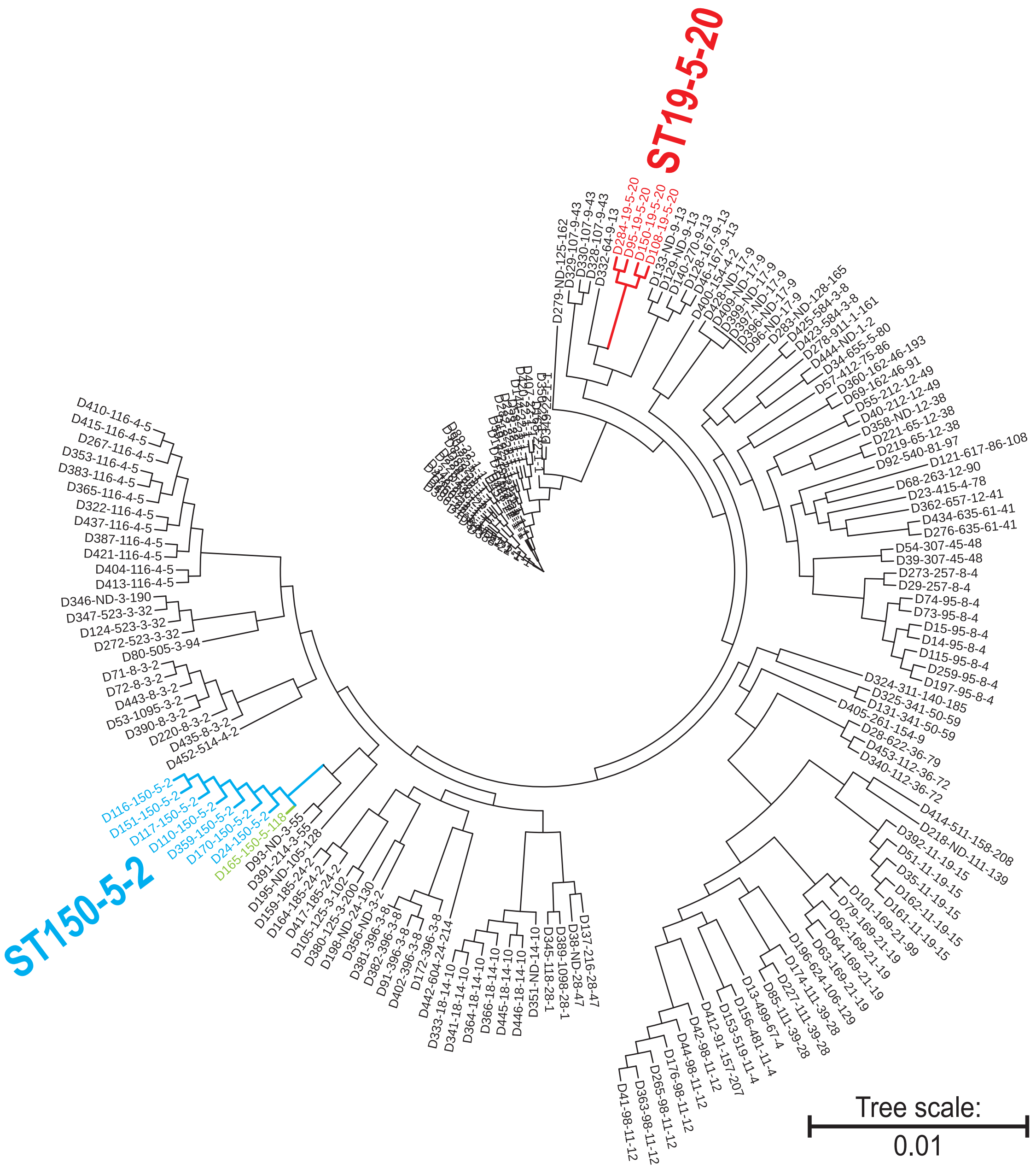

B

Coding #	Accession #	ST	Genotypes of the top seven HND genes.						
			groups_3152	*nanK*	*iprA*	*mipA*	*yehY*	*yhcH*	*ymdB*
D95-19-5-20	CP048416.1	19	5	20	8	45	18	2	1
D150-19-5-20	CP056451.1	19	5	20	8	45	18	2	1
D108-19-5-20	CP055247.1	19	5	20	8	85	18	2	1
D284-19-5-20	CP099303.1	19	5	20	8	122	18	2	1
D24-150-5-2	CP016952.1	150	5	2	12	2	22	1	2
D170-150-5-2	CP056809.1	150	5	2	12	2	22	1	2
D359-150-5-2	CP133060.1	150	5	2	12	2	22	1	2
D110-150-5-2	CP055421.1	150	5	2	12	2	25	1	2
D116-150-5-2	CP055582.1	150	5	2	12	2	25	1	2
D117-150-5-2	CP055588.1	150	5	2	12	2	25	1	2
D151-150-5-2	CP056466.1	150	5	2	12	2	25	1	2
D165-150-5-118	CP056635.1	150	5	118	103	2	22	1	2
D34-655-5-80	CP024672.1	655	5	80	6	5	48	74	9
