# Supplementary material for: Species identification and genotyping of Citrobacter spp. using genes with high nucleotide diversity
Source: Microbiol Spectr. 2026 Apr 16;14(6):e03646-25. doi: 10.1128/spectrum.03646-25 (PMC13228044; doi:10.1128/spectrum.03646-25)
Supplement: Supplemental figures S6 to S10 — Figures S6 to S10. [file spectrum.03646-25-s0003.pdf]

Supplementary Figure S6

A

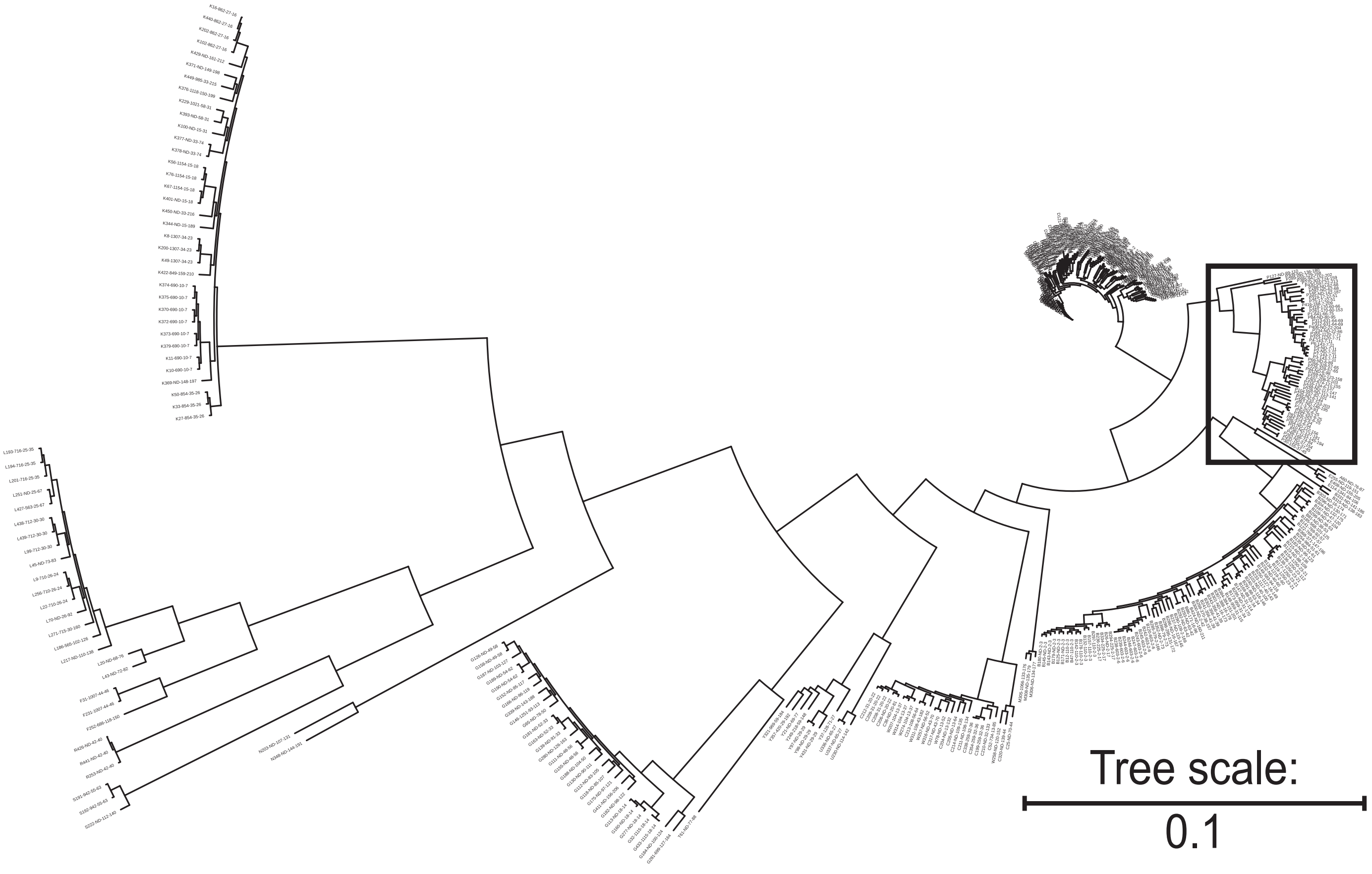

Supplementary Figure S6

B

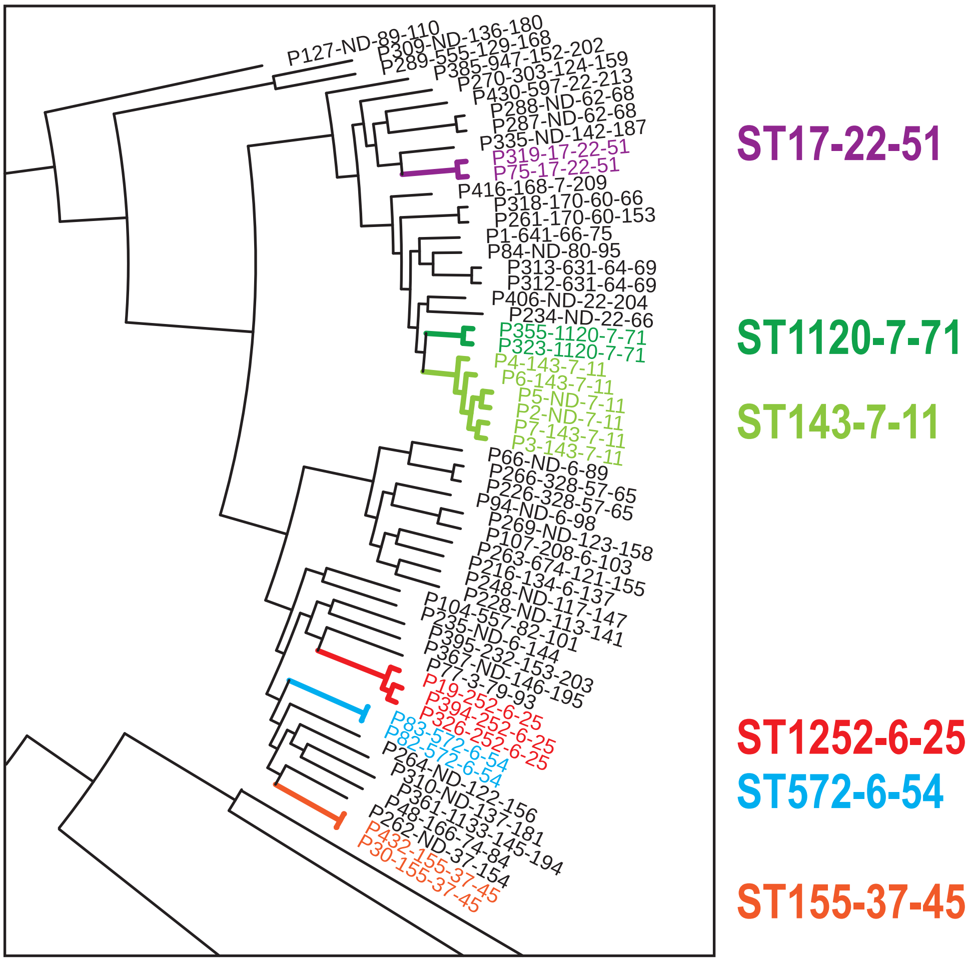

C

			Genotypes of the top seven HND genes.						
Coding #	Accession #	ST	groups_3152	*nanK*	*iprA*	*mipA*	*yehY*	*yhcH*	*ymdB*
P216-134-6-137	CP069801.1	134	6	137	25	101	139	18	116
P107-208-6-103	CP055089.1	208	6	103	29	47	58	18	58
P19-252-6-25	CP012554.1	252	6	25	23	18	28	22	36
P326-252-6-25	CP109744.1	252	6	25	23	18	28	22	36
P394-252-6-25	CP137478.1	252	6	25	23	18	28	22	36
P82-572-6-54	CP045837.1	572	6	54	29	44	54	12	41
P83-572-6-54	CP045840.1	572	6	54	29	44	54	12	41
P66-ND-6-89	CP039327.1	ND	6	89	88	80	30	81	92
P94-ND-6-98	CP048388.1	ND	6	98	25	83	106	18	96
P235-ND-6-144	CP078551.1	ND	6	144	16	108	147	12	41
P3-143-7-11	AP022394.1	143	7	11	3	13	8	8	5
P4-143-7-11	AP022399.1	143	7	11	3	13	8	8	5
P6-143-7-11	AP022494.1	143	7	11	3	13	8	8	5
P7-143-7-11	AP022513.1	143	7	11	3	13	8	8	5
P2-ND-7-11	AP022389.1	ND	7	11	3	13	8	8	5
P5-ND-7-11	AP022486.1	ND	7	11	3	13	8	8	5
P416-168-7-209	CP159117.1	168	7	209	164	7	209	7	54
P323-1120-7-71	CP104921.1	1120	7	71	3	7	81	70	5
P355-1120-7-71	CP126611.1	1120	7	71	3	7	81	70	5
P75-17-22-51	CP043009.1	17	22	51	47	28	31	7	31
P319-17-22-51	CP101100.1	17	22	51	47	28	31	7	31
P430-597-22-213	LR134214.1	597	22	213	70	151	213	147	31
P234-ND-22-66	CP078550.1	ND	22	66	3	7	146	8	49
P406-ND-22-204	CP145154.1	ND	22	204	3	7	204	7	159
P30-155-37-45	CP022311.1	155	37	45	25	39	45	12	55
P432-155-37-45	LR698971.1	155	37	45	25	39	45	12	55
P262-ND-37-154	CP089437.1	ND	37	154	23	115	155	12	127

Supplementary Figure S7

A

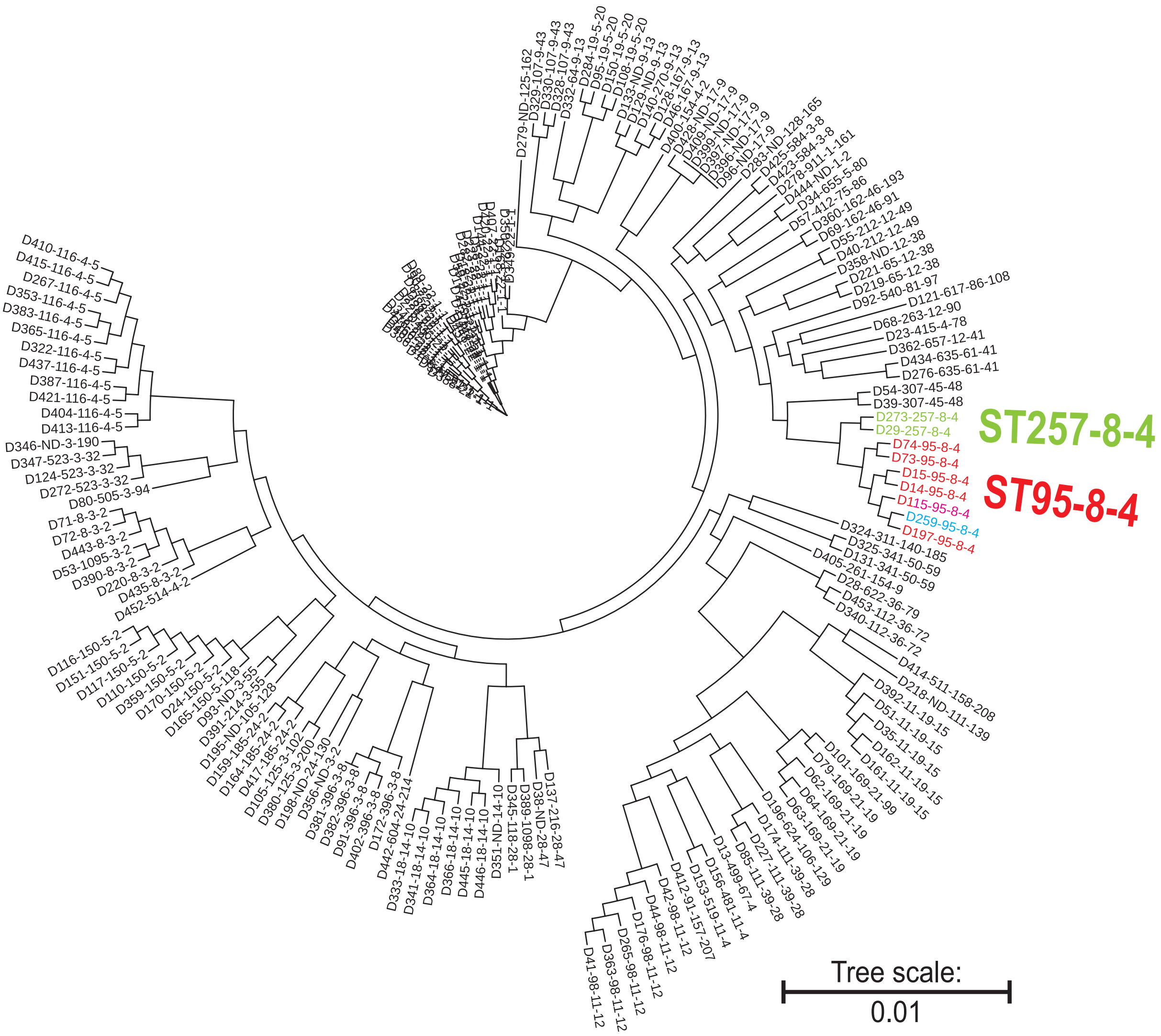

B

Coding #	Accession #	ST	Genotypes of the top seven HND genes.						
			groups_3152	*nanK*	*iprA*	*mipA*	*yehY*	*yhcH*	*ymdB*
D14-95-8-4	AP028314.1	95	8	4	1	10	5	4	8
D15-95-8-4	AP028317.1	95	8	4	1	10	5	4	8
D73-95-8-4	CP042524.1	95	8	4	1	10	5	4	8
D74-95-8-4	CP042534.1	95	8	4	1	10	5	4	8
D115-95-8-4	CP055564.1	95	8	4	1	10	5	4	8
D197-95-8-4	CP059427.1	95	8	4	1	10	5	4	8
D259-95-8-4	CP085726.1	95	8	4	1	114	5	4	8
D29-257-8-4	CP022273.1	257	8	4	1	10	5	4	20
D273-257-8-4	CP097107.1	257	8	4	1	10	5	4	20

Supplementary Figure S8

A

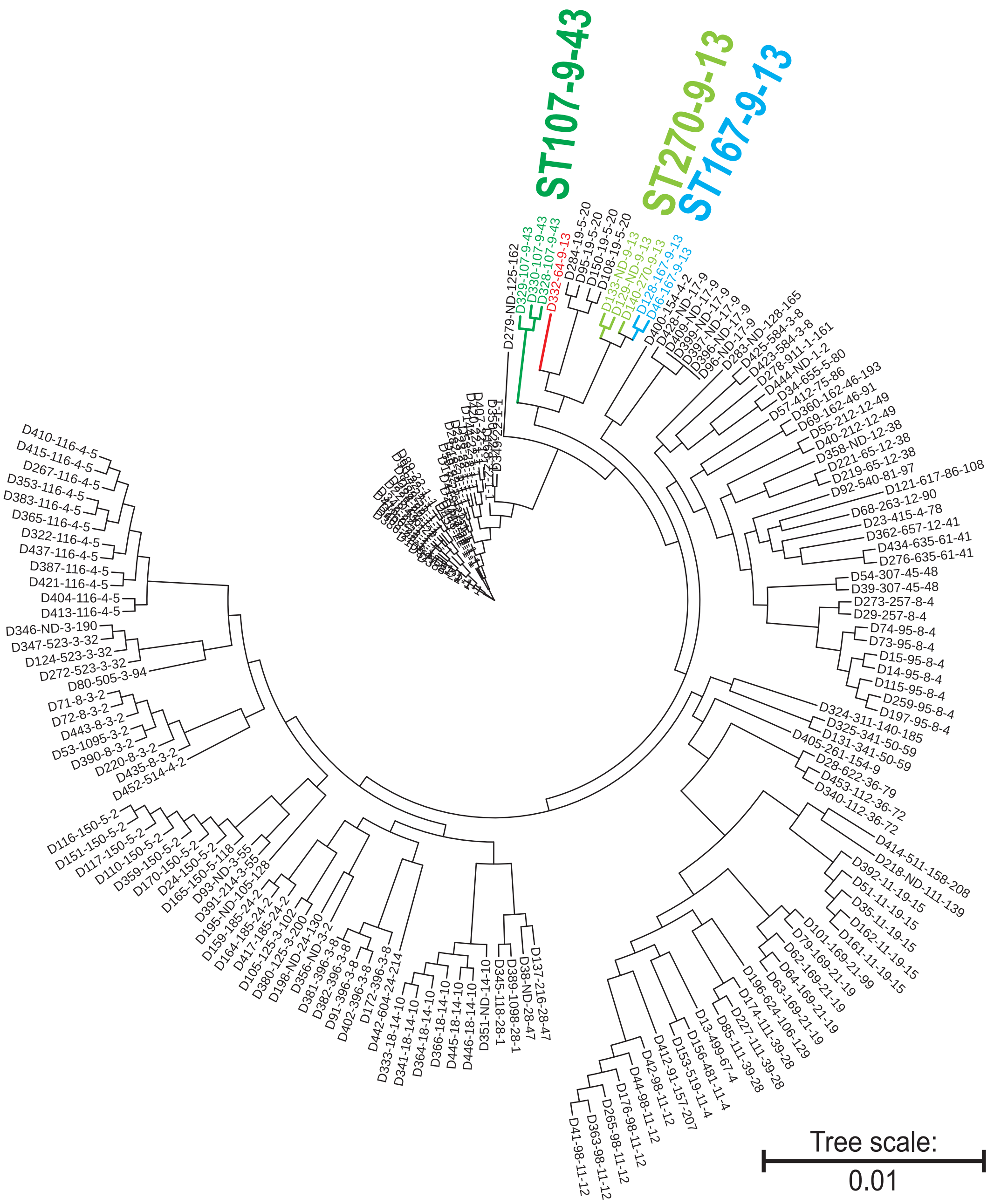

B

			Genotypes of the seven HND genes.						
Coding #	Accession #	ST	groups_3152	*nanK*	*iprA*	*mipA*	*yehY*	*yhcH*	*ymdB*
D332-64-9-13	CP113784.1	64	9	13	8	6	18	1	1
D46-167-9-13	CP026231.1	167	9	13	8	1	10	1	23
D128-167-9-13	CP056235.1	167	9	13	8	1	10	1	23
D140-270-9-13	CP056336.1	270	9	13	8	1	10	1	23
D129-ND-9-13	CP056238.1	ND	9	13	8	49	10	1	23
D133-ND-9-13	CP056256.1	ND	9	13	8	49	10	1	23
D328-107-9-43	CP110894.1	107	9	43	1	2	42	4	53
D329-107-9-43	CP110900.1	107	9	43	1	2	42	4	53
D330-107-9-43	CP110914.1	107	9	43	1	2	42	4	53

Supplementary Figure S9

A

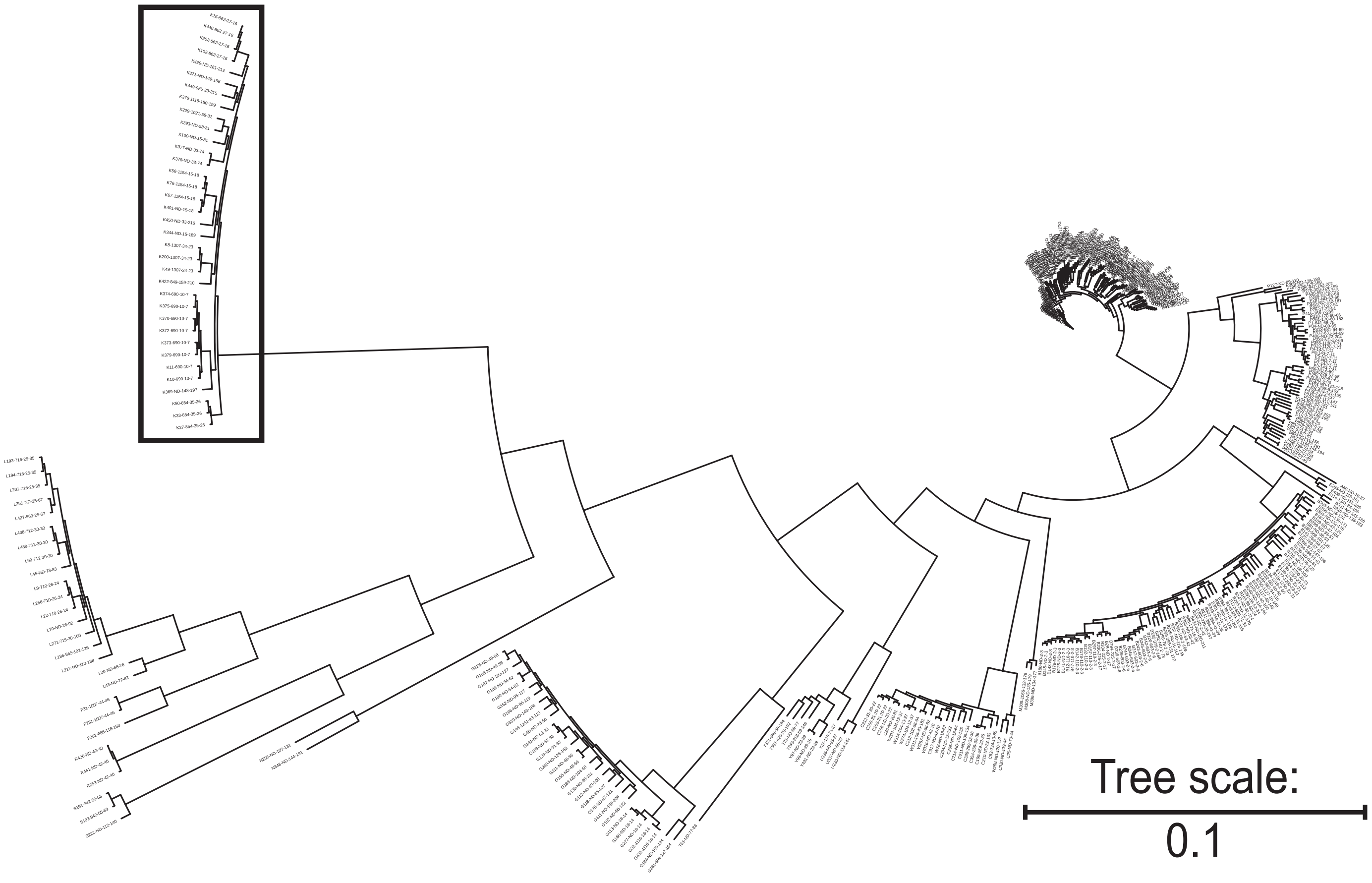

Supplementary Figure S9

B

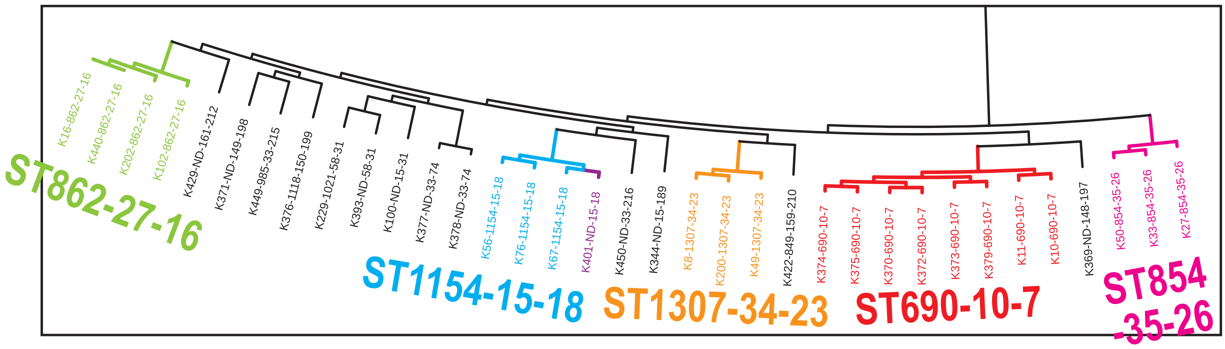

C

			Genotypes of the top seven HND genes.						
Coding	Accession #	ST	groups_3152	*nanK*	*iprA*	*mipA*	*yehY*	*yhcH*	*ymdB*
K10-690-10-7	AP025640.1	690	10	7	11	9	7	11	6
K11-690-10-7	AP025653.1	690	10	7	11	9	7	11	6
K370-690-10-7	CP136808.1	690	10	7	11	9	7	11	6
K372-690-10-7	CP136813.1	690	10	7	11	9	7	11	6
K373-690-10-7	CP136816.1	690	10	7	11	9	7	11	6
K374-690-10-7	CP136819.1	690	10	7	11	9	7	11	6
K375-690-10-7	CP136821.1	690	10	7	11	9	7	11	6
K379-690-10-7	CP137005.1	690	10	7	11	9	7	11	6
K56-1154-15-18	CP033780.1	1154	15	18	26	23	24	20	30
K67-1154-15-18	CP040234.1	1154	15	18	26	23	24	20	30
K76-1154-15-18	CP044097.1	1154	15	18	26	23	24	20	30
K401-ND-15-18	CP139989.1	ND	15	18	26	23	24	20	30
K100-ND-15-31	CP050078.1	ND	15	31	48	84	34	85	32
K344-ND-15-189	CP118927.1	ND	15	189	24	135	186	71	77
K16-862-27-16	CP000822.1	862	27	16	22	22	21	21	26
K102-862-27-16	CP052059.1	862	27	16	22	22	21	21	26
K202-862-27-16	CP066089.1	862	27	16	22	22	21	21	26
K440-862-27-16	NC_009792.1	862	27	16	22	22	21	21	26
K377-ND-33-74	CP136828.1	ND	33	74	69	37	83	19	32
K378-ND-33-74	CP136832.1	ND	33	74	69	37	83	19	32
K449-985-33-215	OW969691.1	985	33	215	169	37	216	149	79
K450-ND-33-216	OW969711.1	ND	33	216	170	153	217	20	77
K8-1307-34-23	AP023452.1	1307	34	23	31	14	20	30	34
K49-1307-34-23	CP026697.1	1307	34	23	31	14	20	30	34
K200-1307-34-23	CP060484.1	1307	34	23	31	14	20	30	34
K27-854-35-26	CP022073.2	854	35	26	24	14	29	19	37
K33-854-35-26	CP023527.1	854	35	26	24	14	29	19	37
K50-854-35-26	CP026709.1	854	35	26	24	14	29	19	37

Supplementary Figure S10

A

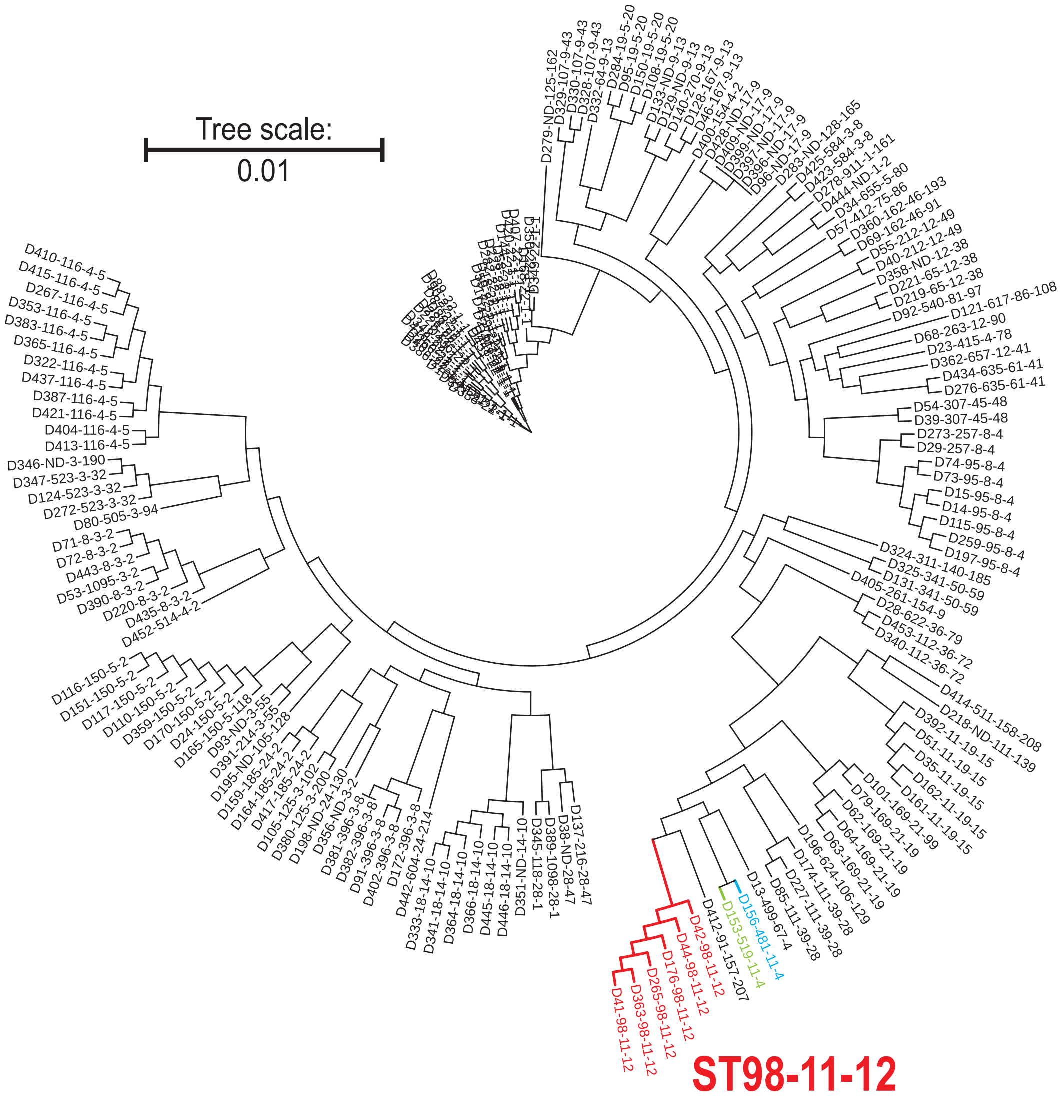

B

			Genotypes of the top seven HND genes.						
Coding	Accession #	ST	groups_ 3152	*nanK*	*iprA*	*mipA*	*yehY*	*yhcH*	*ymdB*
D42-98-11-12	CP024881.1	98	11	12	1	73	9	9	18
D44-98-11-12	CP026056.1	98	11	12	1	1	9	9	18
D176-98-11-12	CP056852.1	98	11	12	1	1	9	9	18
D265-98-11-12	CP092463.1	98	11	12	1	1	9	9	18
D363-98-11-12	CP135465.1	98	11	12	1	1	9	9	18
D41-98-11-12	CP024683.1	98	11	12	78	1	9	9	18
D156-481-11-4	CP056527.1	481	11	4	56	1	65	5	63
D153-519-11-4	CP056505.1	519	11	4	56	1	65	5	63
