# Supplementary material for: Species identification and genotyping of Citrobacter spp. using genes with high nucleotide diversity
Source: Microbiol Spectr. 2026 Apr 16;14(6):e03646-25. doi: 10.1128/spectrum.03646-25 (PMC13228044; doi:10.1128/spectrum.03646-25)

Supplementary Figure S11

A

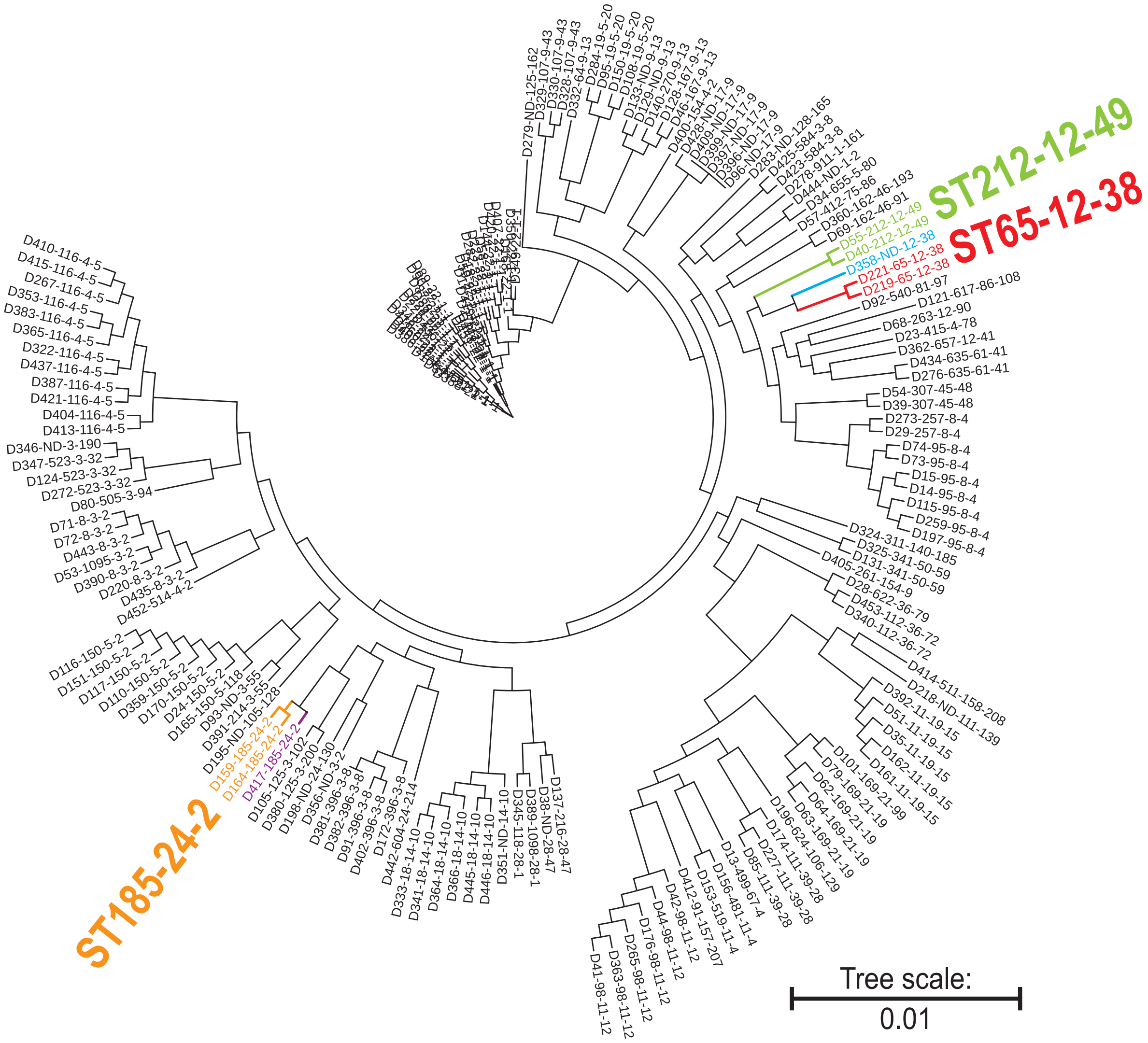

B

			Genotypes of the top seven HND genes.						
Coding	Accession #	ST	groups_3152	*nanK*	*iprA*	*mipA*	*yehY*	*yhcH*	*ymdB*
D219-65-12-38	CP070544.1	65	12	38	60	27	72	2	68
D221-65-12-38	CP070559.1	65	12	38	60	27	72	2	68
D358-ND-12-38	CP132324.1	ND	12	38	1	138	191	2	148
D40-212-12-49	CP024680.1	212	12	49	77	42	51	24	57
D55-212-12-49	CP033744.1	212	12	49	84	42	51	24	57
D68-263-12-90	CP040698.1	263	12	90	6	27	99	25	20
D362-657-12-41	CP135450.1	657	12	41	1	139	193	25	150
D159-185-24-2	CP056573.1	185	24	2	1	6	66	1	7
D164-185-24-2	CP056622.1	185	24	2	1	6	66	1	7
D417-185-24-2	CP162039.1	185	24	2	1	6	210	1	7
D442-604-24-214	OW848788.1	604	24	214	6	152	3	148	7
D198-ND-24-130	CP059849.1	ND	24	130	1	20	132	2	111

Supplementary Figure S12

A

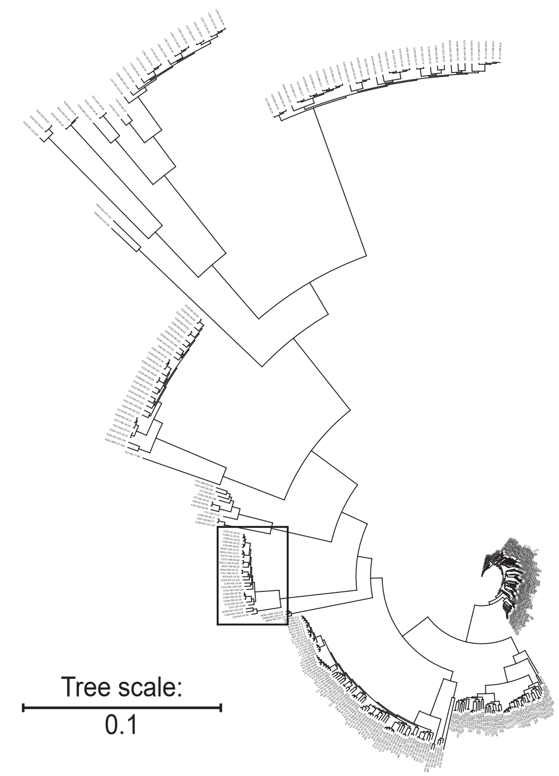

B

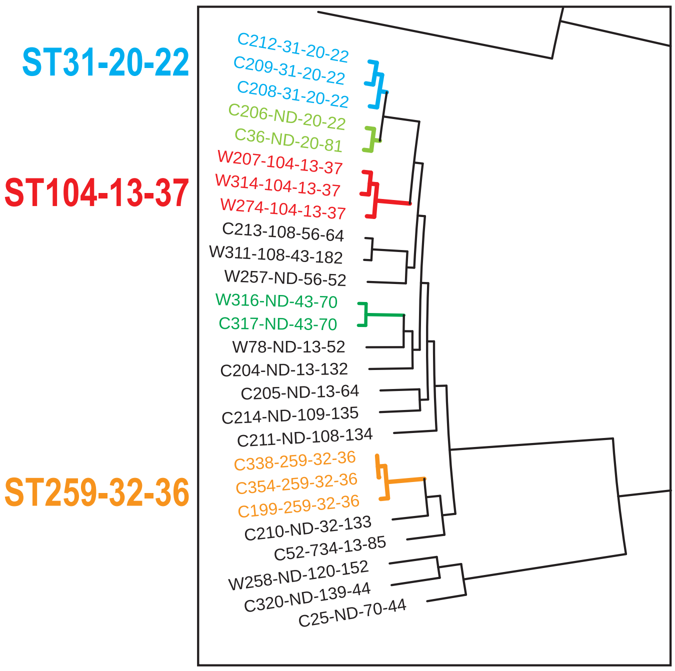

C

			Genotypes of the top seven HND genes.						
Coding	Accession #	ST	groups_ 3152	*nanK*	*iprA*	*mipA*	*yehY*	*yhcH*	*ymdB*
W207-104-13-37	CP069770.1	104	13	37	21	34	39	13	114
W274-104-13-37	CP097324.1	104	13	37	21	34	39	13	13
W314-104-13-37	CP101080.1	104	13	37	21	34	39	13	13
C52-734-13-85	CP029727.1	734	13	85	83	77	94	77	88
W78-ND-13-52	CP044101.1	ND	13	52	89	82	102	13	13
C205-ND-13-64	CP069764.1	ND	13	64	116	53	134	13	113
C204-ND-13-132	CP069763.1	ND	13	132	115	26	70	13	13
C208-31-20-22	CP069779.1	31	20	22	15	19	14	17	17
C209-31-20-22	CP069781.1	31	20	22	15	19	14	17	17
C212-31-20-22	CP069784.1	31	20	22	15	19	14	17	17
C206-ND-20-22	CP069768.1	ND	20	22	15	19	14	17	17
C36-ND-20-81	CP024675.1	ND	20	81	15	19	14	17	17
C199-259-32-36	CP060441.1	259	32	36	38	26	38	28	33
C338-259-32-36	CP115032.1	259	32	36	38	26	38	28	33
C354-259-32-36	CP126605.1	259	32	36	38	26	38	28	33
C210-ND-32-133	CP069782.1	ND	32	133	117	98	135	28	33
W311-108-43-182	CP101066.1	108	43	182	59	54	71	29	13
W316-ND-43-70	CP101089.1	ND	43	70	21	65	80	29	75
C317-ND-43-70	CP101092.1	ND	43	70	21	65	80	29	75

Supplementary Figure S13

A

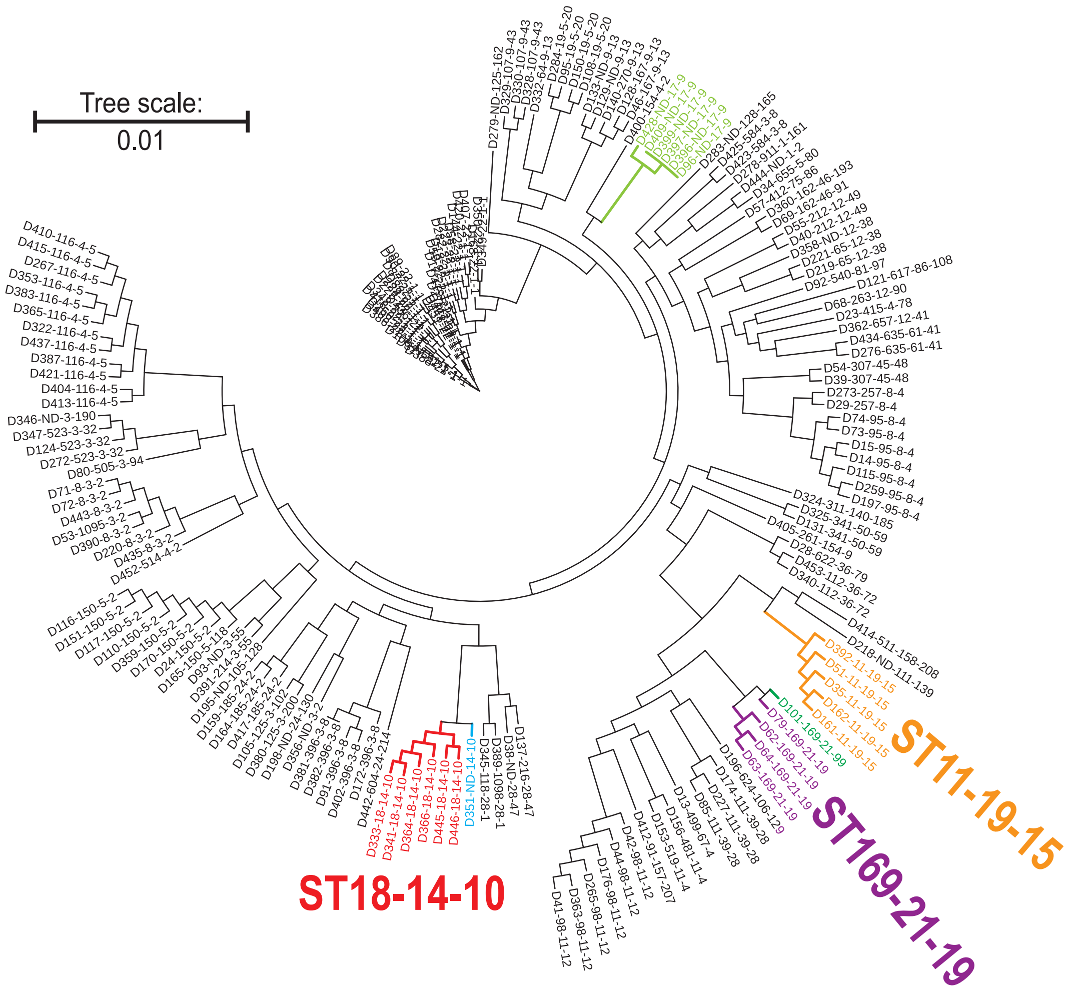

B

			Genotypes of the top seven HND genes.						
Coding	Accession #	ST	groups_3152	*nanK*	*iprA*	*mipA*	*yehY*	*yhcH*	*ymdB*
D333-18-14-10	CP114564.1	18	14	10	6	2	3	14	16
D341-18-14-10	CP115614.1	18	14	10	6	2	3	14	16
D364-18-14-10	CP135469.1	18	14	10	6	2	3	14	16
D366-18-14-10	CP136047.1	18	14	10	6	2	3	14	16
D445-18-14-10	OW849208.1	18	14	10	6	2	3	14	16
D446-18-14-10	OW849256.1	18	14	10	6	2	3	14	16
D351-ND-14-10	CP125310.1	ND	14	10	6	2	189	14	16
D96-ND-17-9	CP049015.1	ND	17	9	9	2	12	15	19
D396-ND-17-9	CP137703.1	ND	17	9	9	2	12	15	19
D397-ND-17-9	CP137717.1	ND	17	9	9	2	12	15	19
D399-ND-17-9	CP139745.1	ND	17	9	9	2	12	15	19
D409-ND-17-9	CP149135.1	ND	17	9	9	2	12	15	19
D428-ND-17-9	LR134118.1	ND	17	9	166	2	12	15	19
D35-11-19-15	CP024673.1	11	19	15	14	1	13	5	22
D161-11-19-15	CP056592.1	11	19	15	14	1	13	5	22
D162-11-19-15	CP056595.1	11	19	15	14	1	13	5	22
D392-11-19-15	CP137207.1	11	19	15	14	1	13	5	22
D51-11-19-15	CP027849.1	11	19	15	82	1	13	5	22
D62-169-21-19	CP038653.1	169	21	19	27	1	15	1	11
D63-169-21-19	CP038656.1	169	21	19	27	1	15	1	11
D64-169-21-19	CP038658.1	169	21	19	27	1	15	1	11
D79-169-21-19	CP045555.1	169	21	19	90	1	15	1	11
D101-169-21-99	CP052058.1	169	21	99	27	1	15	1	11

Supplementary Figure S14

A

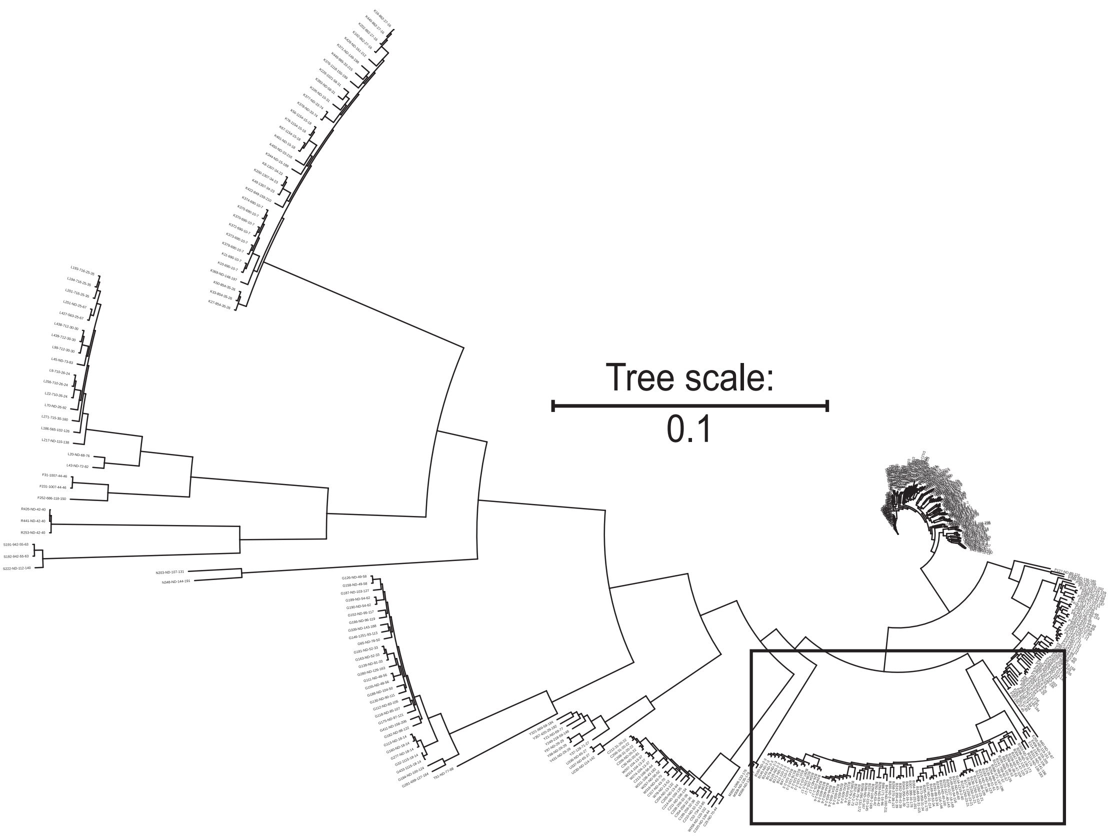

Supplement: Supplemental figures S11 to S14 — Figures S11 to S14. [file spectrum.03646-25-s0004.pdf]
