# Supplementary material for: Species identification and genotyping of Citrobacter spp. using genes with high nucleotide diversity
Source: Microbiol Spectr. 2026 Apr 16;14(6):e03646-25. doi: 10.1128/spectrum.03646-25 (PMC13228044; doi:10.1128/spectrum.03646-25)
Supplement: Supplemental figures S14 to S17 — Figures S14 to S17. [file spectrum.03646-25-s0005.pdf]

Supplementary Figure S14

B

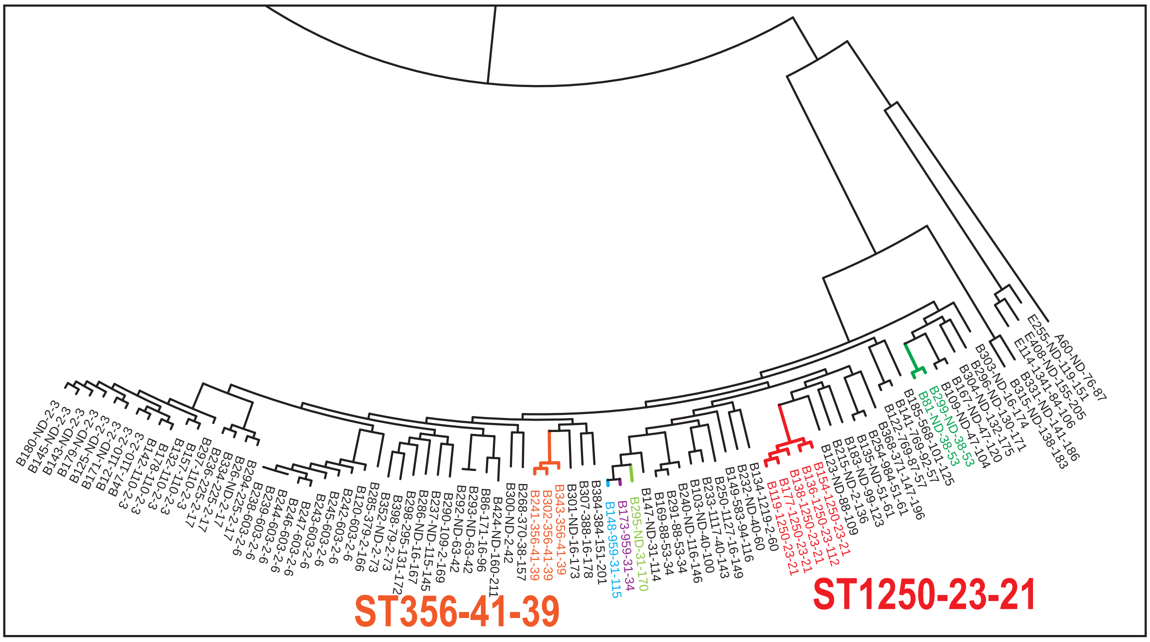

C

			Genotypes of the top seven HND genes.						
Coding	Accession #	ST	groups_ 3152	*nanK*	*iprA*	*mipA*	*yehY*	*yhcH*	*ymdB*
B86-171-16-96	CP046673.1	171	16	96	17	4	103	84	94
B307-388-16-178	CP101044.1	388	16	178	142	62	176	41	14
B250-1127-16-149	CP080539.1	1127	16	149	10	112	151	114	124
B286-ND-16-167	CP099366.1	ND	16	167	7	4	166	41	14
B301-ND-16-173	CP099387.1	ND	16	173	63	125	171	42	3
B303-ND-16-174	CP099389.1	ND	16	174	140	126	172	126	14
B119-1250-23-21	CP055910.1	1250	23	21	19	21	19	16	25
B138-1250-23-21	CP056321.1	1250	23	21	19	21	19	16	25
B154-1250-23-21	CP056508.1	1250	23	21	19	21	19	16	25
B177-1250-23-21	CP056861.1	1250	23	21	19	21	19	16	25
B136-1250-23-112	CP056284.1	1250	23	112	19	21	19	16	25
B148-959-31-115	CP056409.1	959	31	115	10	32	26	27	46
B173-959-31-34	CP056834.1	959	31	34	10	32	26	27	46
B295-ND-31-170	CP099379.1	ND	31	170	10	32	26	16	14
B147-ND-31-114	CP056399.1	ND	31	114	10	51	118	57	46
B268-370-38-157	CP092860.1	370	38	157	13	118	158	16	130
B81-ND-38-53	CP045771.1	ND	38	53	28	43	53	50	40
B299-ND-38-53	CP099385.1	ND	38	53	138	43	53	50	40
B233-1117-40-143	CP077405.1	1117	40	143	49	11	56	63	121
B103-ND-40-100	CP053573.1	ND	40	100	49	11	56	86	14
B232-ND-40-60	CP077300.1	ND	40	60	10	107	145	112	120
B241-356-41-39	CP078598.1	356	41	39	30	35	40	39	50
B302-356-41-39	CP099388.1	356	41	39	30	35	40	39	50
B343-356-41-39	CP118774.1	356	41	39	30	35	40	39	50

Supplementary Figure S15

A

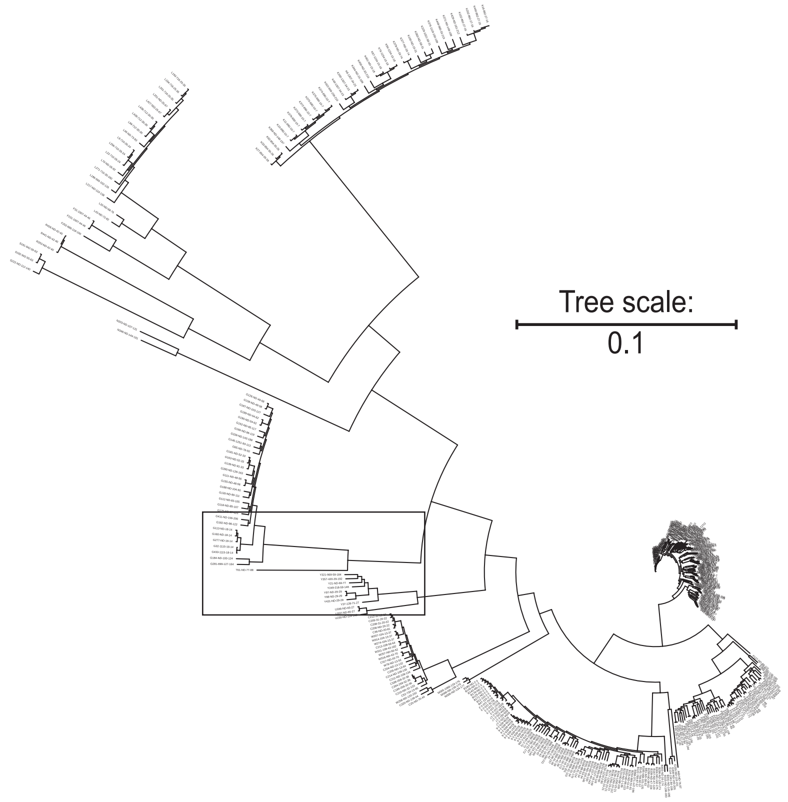

B

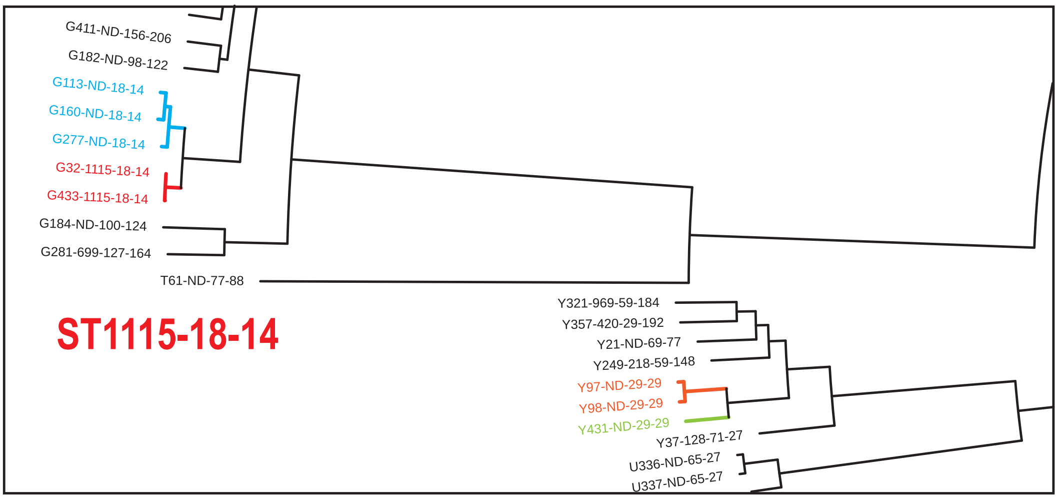

C

			Genotypes of the top seven HND genes.						
Coding	Accession #	ST	groups_ 3152	*nanK*	*iprA*	*mipA*	*yehY*	*yhcH*	*ymdB*
G32-1115-18-14	CP023504.1	1115	18	14	42	41	47	47	21
G433-1115-18-14	LR699014.1	1115	18	14	42	41	47	47	21
G113-ND-18-14	CP055543.1	ND	18	14	51	31	35	34	21
G160-ND-18-14	CP056586.1	ND	18	14	51	31	35	34	21
G277-ND-18-14	CP099084.1	ND	18	14	133	31	35	34	21
Y357-420-29-192	CP132279.1	420	29	192	40	137	190	44	70
Y97-ND-29-29	CP049738.1	ND	29	29	33	24	55	52	27
Y98-ND-29-29	CP049739.1	ND	29	29	33	24	55	52	27
Y431-ND-29-29	LR134485.1	ND	29	29	33	24	214	65	27

Supplementary Figure S16

A

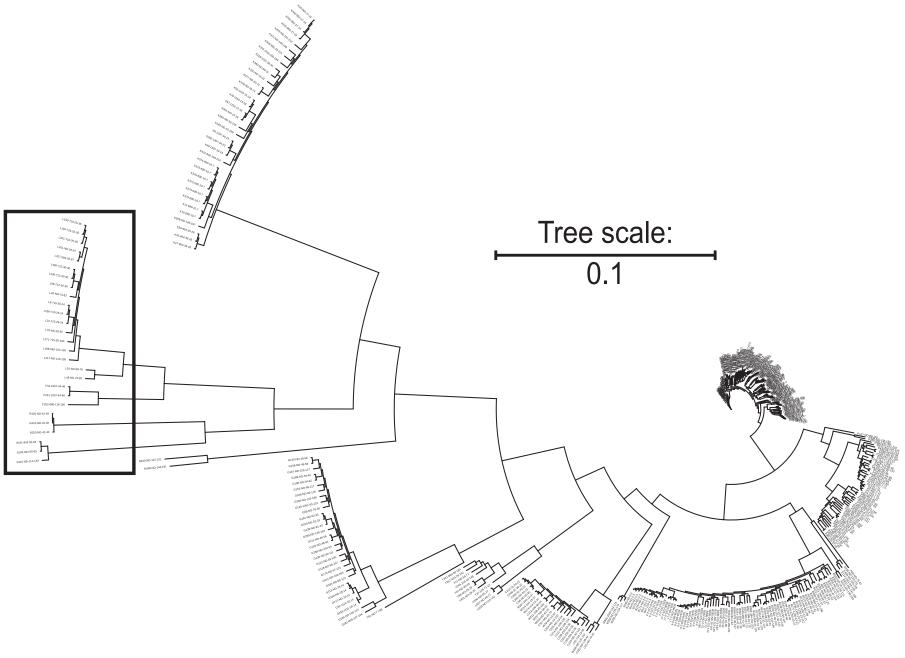

B

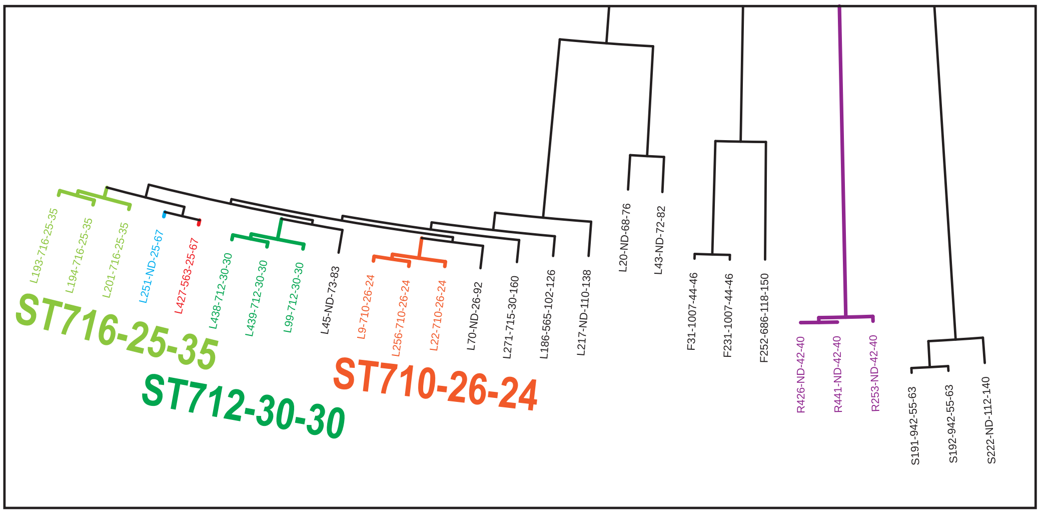

C

			Genotypes of the top seven HND genes.						
Coding	Accession #	ST	groups_ 3152	*nanK*	*iprA*	*mipA*	*yehY*	*yhcH*	*ymdB*
L427-563-25-67	JASWFH010000001.1	563	25	67	20	15	73	66	12
L251-ND-25-67	CP080958.1	ND	25	67	20	15	73	66	12
L193-716-25-35	CP057630.1	716	25	35	20	15	37	38	12
L194-716-25-35	CP057632.1	716	25	35	20	15	37	38	12
L201-716-25-35	CP064180.1	716	25	35	20	15	37	38	12
L22-710-26-24	CP014070.2	710	26	24	32	15	27	31	35
L9-710-26-24	AP024585.1	710	26	24	32	38	27	31	35
L256-710-26-24	CP083651.1	710	26	24	32	38	27	31	35
L70-ND-26-92	CP041362.1	ND	26	92	46	81	100	82	12
L99-712-30-30	CP050009.1	712	30	30	34	29	33	33	44
L438-712-30-30	LT556084.1	712	30	30	34	29	33	33	44
L439-712-30-30	LT556085.1	712	30	30	34	29	33	33	44
L271-715-30-160	CP096905.1	715	30	160	46	120	160	119	12
R253-ND-42-40	CP082833.1	ND	42	40	39	36	41	40	51
R426-ND-42-40	FN543502.1	ND	42	40	39	36	41	40	51
R441-ND-42-40	NC_013716.1	ND	42	40	39	36	41	40	51

Supplementary Figure S17

A

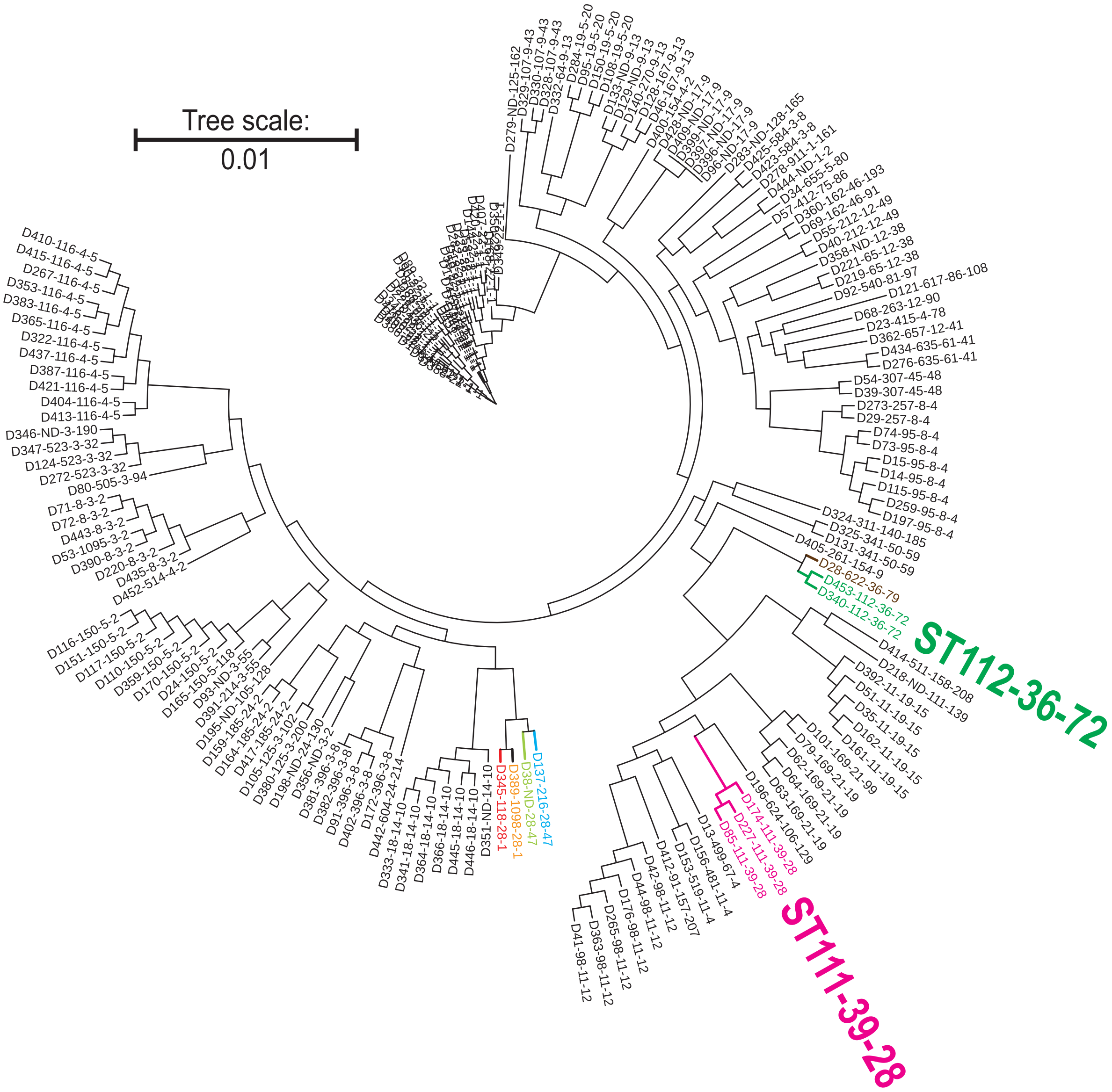

B

			Genotypes of the top seven HND genes.						
Coding	Accession #	ST	groups_3152	*nanK*	*iprA*	*mipA*	*yehY*	*yhcH*	*ymdB*
D345-118-28-1	CP119048.1	118	28	1	37	1	3	1	29
D389-1098-28-1	CP137195.1	1098	28	1	37	1	3	1	29
D137-216-28-47	CP056314.1	216	28	47	37	1	116	2	29
D38-ND-28-47	CP024677.1	ND	28	47	76	1	49	2	29
D340-112-36-72	CP115129.1	112	36	72	1	6	44	9	38
D453-112-36-72	OW995941.1	112	36	72	1	6	218	9	38
D28-622-36-79	CP022151.1	622	36	79	1	6	44	9	38
D85-111-39-28	CP046502.1	111	39	28	1	1	32	5	43
D174-111-39-28	CP056839.1	111	39	28	1	1	32	5	43
D227-111-39-28	CP073043.1	111	39	28	1	1	32	5	43
